# Reactivity of 4,5-Dichlorophthalic Anhydride towards Thiosemicarbazide and Amines: Synthesis, Spectroscopic Analysis, and DFT Study

**DOI:** 10.3390/molecules27113550

**Published:** 2022-05-31

**Authors:** Hatem A. Abuelizz, Ahmed H. Bakheit, Mohamed Marzouk, Mohamed M. Abdellatif, Rashad Al-Salahi

**Affiliations:** 1 Department of Pharmaceutical Chemistry, College of Pharmacy, King Saud University, Riyadh 11451, Saudi Arabia; abujazz76@gmail.com; 2 Chemistry of Tanning Materials and Leather Technology Department, Chemical Industries Research Institute, National Research Centre, 33 El-Bohouth St. (Former El-Tahrir St.), Dokki, Cairo 12622, Egypt; msmarzouk@yahoo.co.uk; 3 Department of Chemistry, Graduate School of Science, Tokyo Metropolitan University, 1-1 Minami Osawa, Tokyo 192-0397, Japan; mohamed-soliman@tmu.ac.jp

**Keywords:** 4,5-Dichlorophthalic anhydride, aldehydes, thiosemicarbazide, amines, DFT, IR, NMR, Fukui functions

## Abstract

The cyclic anhydrides are broadly employed in several fields, such as the chemical, plastic, agrochemical, and pharmaceutical industries. This study describes the chemical reactivity of 4,5-dichlorophthalic anhydride towards several nucleophiles, including thiosemicarbazide and different amines, to produce the carboxylic acid derivatives resulting from anhydride’s opening, namely, phthalimide and dicarboxylic acid (**1**–**12**) products. Their chemical structures are confirmed by NMR, IR and MS spectra analyses. Density–functional theory (DFT) studies are performed using (DFT/B3LYP) with the 6-311G(d, p) basis sets to recognize different chemical and physical features of the target compounds.

## 1. Introduction

**Figure 1:**
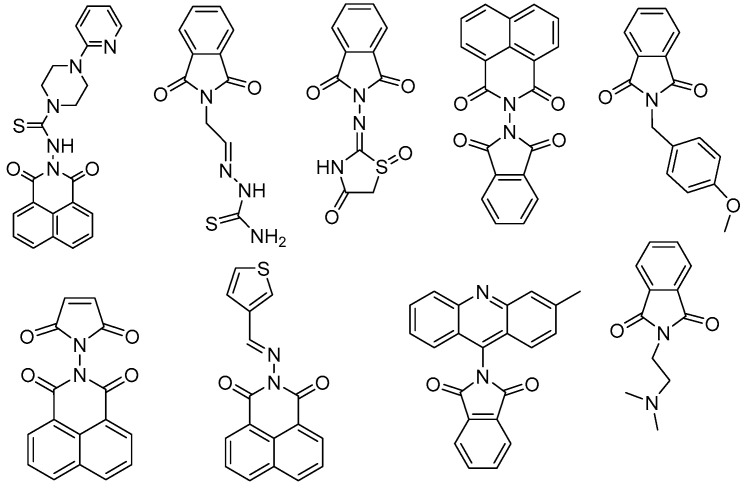
The reported bioactive cyclic anhydrides.

Synthesis of new chemical compounds and evaluation of their biological properties are the main tasks in organic medicinal chemistry research. A literature survey revealed that several heterocyclic compounds originating from cyclic acid anhydrides have made a great contribution in a number of interesting areas [1,2,3,4,5,6,7]. Cyclic acid anhydrides (phthalic, naphthalic, pyromellitic, and maleic acids, etc.) are considered to be building blocks in the construction of many scaffolds with different applications in chemical and pharmaceutical fields. For instance, they are essential in plastic synthetic resins (glyptal, alkyd, polyester), polyurethane, household appliances, and medical device coatings, and are used as key intermediates in phthalein, rhodamine, phthalocyanine, anthraquinone, fluorescein, and agrochemical production [1,2,3,4,5,6,7]. Moreover, many cyclic anhydride derivatives participate in the building of additional bioactive compounds with plethora of biological activities, including antiviral, antitumor, immunomodulatory, cytotoxic, and antimicrobial agents [3,4,5,6,7,8,9,10,11,12,13,14,15,16,17,18,19,20] (Figure 1). Phthalic anhydride, known as 2-benzofuran-1,3-dione, is characterized by oxygen-rich atoms and was first made commercially available as dicarboxylic acid anhydride. It is synthesized from phthalic acid dehydration reaction or can be obtained via Diels–Alder cycloaddition of furan and maleic anhydride followed by dehydration [2,18]. Phthalic anhydrides are widely used in the chemical industry, with one major application in the production of several phthalate esters which are widely used in the plastics industry as plasticizers. Moreover, they are utilized in dye formation as quinizarin pigment as well as phenolphthalein. The coordination behavior of phthalic anhydride ligands with metal atoms has been studied, providing important information about their co-ordination chemistry and biological applications [6,18]. 

Furthermore, phthalic anhydrides are characterized by dominant saturated dibasic acids with a high chemical affinity towards different nucleophiles (thiosemicarbazide, amines, alcohols, hydroxyl amines, etc.), affording the carboxylic acid derivatives arising from anhydride opening, namely, phthalimides, dicarboxylic acids and carboxylic acid esters. Considering both the above facts and the continuing work in chemical research dealing with cyclic anhydrides, the present work aimed to synthesize compounds **1**–**12** starting with 4,5-dichlorophthalic as starting material. The chemical structures of the synthesized compounds **1**–**12** were confirmed using different spectroscopic analytical tools, i.e., MS, IR, and NMR. Density function theory (DFT) calculations were carried out based on set (B3LYP) with the 6-311G(d, p) level of theory. To clarify the chemical and physical characteristics of the target compounds **1**–**12**, various descriptors such as ionization potential (*IP*), the electron affinity (*EA*), chemical potential (*μ*), electronegativity (*χ*), hardness (*η*), softness (*S*), electrophilicity index (*ω*), and nucleophilicity index (*N*) were calculated.

## 2. Results and Discussion

### 2.1. Chemistry

**Scheme 1:**
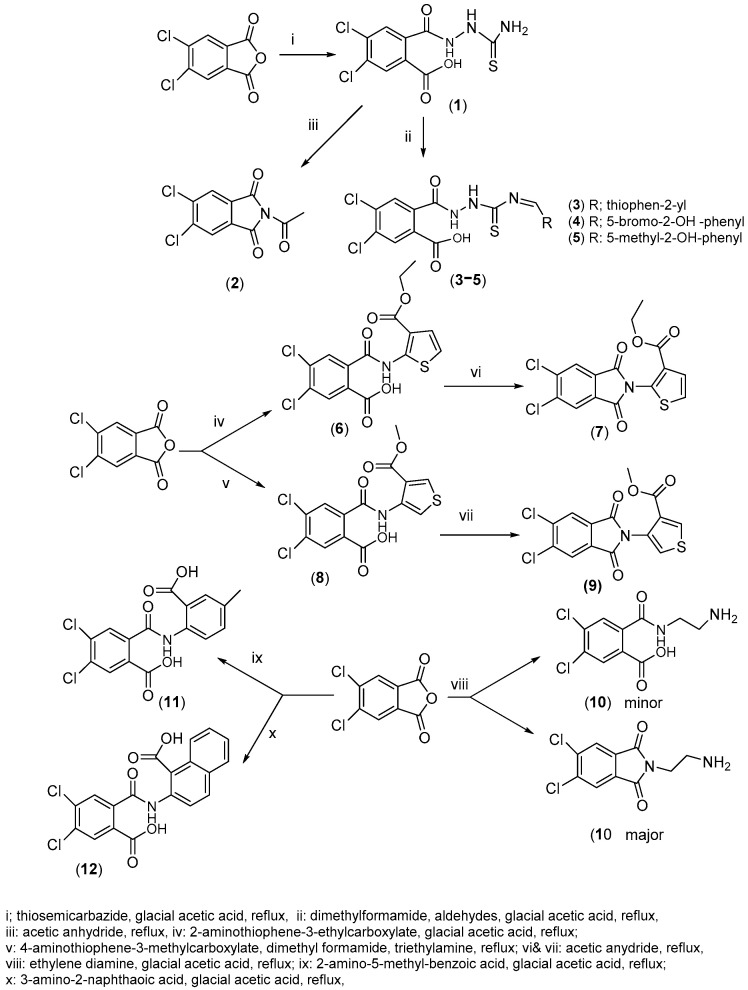
Synthetic routes for compounds **1**–**12.**

Many reactions are aimed at, as indicated by Scheme 1, to afford the products **1**–**12** using 4,5-dichlorophthalic anhydride as key intermediate. The 4,5-dichlorophthalic anhydride was dissolved in boiling glacial acetic acid and treated with thiosemicarbazide under reflux condition to obtain the parent compound **1**, with a good yield [9,16]. The structural identity of **1** was fully characterized via interpretation of its IR, MS, and NMR spectral data. The IR spectrum of compound **1** revealed stretching absorption bands at 1663 and 1702 cm^−1^ for carbonyl groups and weak absorption bands in the range of 3243–3439 cm^−1^ for amino groups (see Supplementary Figure). The EI-HRMS spectrum showed the molecular radical cation peaks at *m*/*z* 306.9503 (see Experimental section), which corresponds to a molecular formula of C_9_H_7_Cl_2_N_3_O_3_S for **1**.

The confirmation of successful synthesis of the target compounds was achieved via interpretation of their ^1^H and ^13^C NMR spectral data. In the aromatic region of the ^1^H NMR spectrum, compound **1** showed two singlets at *δ* ppm 8.22 (H-6) and 8.07 (H-3) for the *ortho* 4,5-dichlorophenyl moiety (see Supplementary Figure). The ^1^H spectrum showed the most corresponding downfield singlets of all exchangeable protons i.e., -NH-, -NH_2_, or COOH (see experimental data), whereas the ^13^C NMR spectrum exhibited a thioxo carbon resonance at 182.7 ppm (see Supplementary Figure) along with another two carbonyl-carbons in the range of 166 and 166.5, which can be interpreted as carboxyl- and imide carbonyls in order to confirm the structure of **1**.

The corresponding compound **2** was obtained with a good yield when the parent **1** was refluxed in acetic anhydride for 7 hrs (Scheme 1). The IR spectrum of **2** clearly showed the absorption stretching frequencies of the carbonyl groups at 1727 and 1796 cm^−1^ (see Supplementary Figure) and confirmed the disappearance of the –NH frequencies in the range of 3243–3439 cm^−1^. A symmetrical structure for the 1,3-dione **2** was suggested first from a ^1^H NMR spectrum that showed a singlet of the two equivalent protons (H-4/7) at 8.50 ppm and then from the ^13^C, which exhibited its ^13^C-resonance at 127.2 (C-4/7). Further, a characteristic resonance was recorded at 163.3 ppm, assignable to the two equivalent dione carbons (C-1/3). The *N*-acetyl group was proven from its ^1^H-singlet at 2.43 (3H, −NCOCH_3_) and ^13^C-signals at 170.0 and 24.7, corresponding to the carbonyl and methyl carbons, respectively (see Supplementary Figures). Reaction of equimolar amounts of **1** and appropriate aldehydes (thiophen-2-carbaldehyde, 5-bromo-2-hydroxybenzaldehyde, and 2-hydroxy-5-methylbenzaldehyde) in boiling DMF produced compounds **3**–**5**. Their IR spectra revealed absorption peaks in the ranges of 1689–1703 and 3367–3405 cm^−1^, indicative of the carbonyl and amino functional groups, respectively. Products **3**–**5** were established on the basis of the characteristic signals of 4,5-dichlorobenzoic acid and corresponding aldehyde together with the two most downfield singlets at about 11.42 and 10.20, assignable to the –NH protons in -CO.NH.NH.CS- group. ^1^H NMR spectra revealed the two singlets typical of H-6 and H-3 at almost 8.21 and 8.16 of the 4,5-dichlorobenzoic acid moiety (**4**), alongside the intrinsic –N=CH- singlet at about 8.33 ppm. Three further informative ^1^H-signals that described the carbaldehyde moieties were unambiguously assigned (see experimental data). The most characteristic ^13^C-resonances in case of such products (**3**–**5**) were assigned at the ranges of 178.0–179.0 and 155.0–156.0 ppm, interpretable as C=S and –N=CH- carbons, respectively. Two other confirmative ^13^C-signals for all 4,5-dichloro derivatives were assigned at about 138.0 and 135.0 for the two chlorinated carbons C-4 and C-5. In regard to the NMR spectrum of compound **5**, it revealed a singlet at 2.22 ppm, assignable to the methyl group and its characteristic ^13^C-resonance at 20.45 ppm (see Supplementary Figures). As illustrated in Scheme 1, reaction of 4,5-dichlorophthalic anhydride with 2-amino-thiophene-3-carboxylic acid ethyl ester in boiling glacial acetic acid smoothly afforded compound **6**, whereas its reaction with 4-aminothiophene-3-methylcarboxylate hydrochloride required a basic medium and proceeded in DMF to obtain the target compound **8**. The IR spectra for compounds **6** and **8** are characterized by strong stretching absorption peaks in the range of 1656−1730 cm^−1^ for carbonyl groups and weak ones at the range of 3270−3400 cm^−1^, assignable to amino groups. Further confirmation for **6** and **8** was carried out via EI-HRMS, where the molecular ion peaks appeared at *m*/*z* 386.9694 (see Supplementary Figure) and 372.9612 for **6** and **8**, respectively.

**Figure 2:**
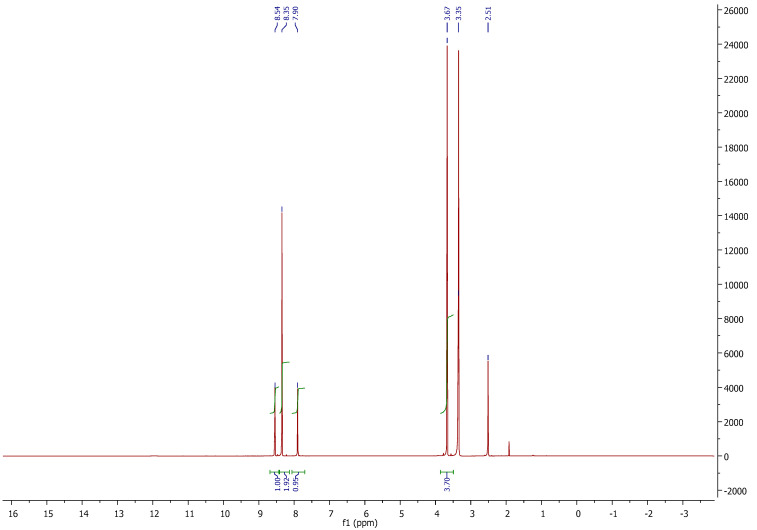
^1^H NMR spectrum (700 MHz, DMSO-*d*_6_) of compound **9**.

**Figure 3:**
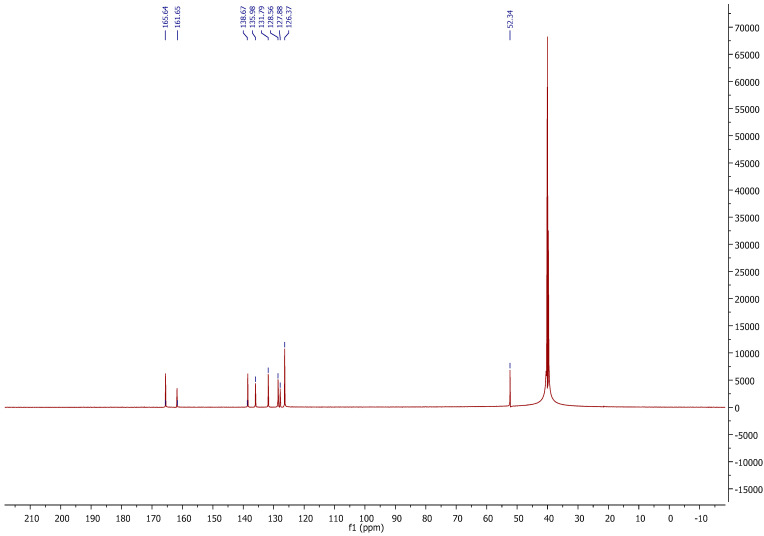
^13^C NMR spectrum (175 MHz, DMSO-*d*_6_) of compound **9**.

The formation of the two coupling products (**6** and **8**) was established by IR, NMR, and HRMS analytical techniques. As in the case of products **3**–**5**, both **6** and **8** showed the two characteristic singlets in the range of 8.1–8.0 ppm in their ^1^H NMR spectra, indicative of H-6 and H-3. In addition, the spectrum of **6** exhibited the characteristic broad singlets at 7.23 (H-5’) and 7.13 (H-4’) together with the ethyl ester resonances at 4.29 (q, -CH_2_CH_3_) and 1.29 (t, -CH_2_CH_3_) (see Supplementary Figures). However, derivative **8** revealed its characteristic thiophene protons as a multiplet at 7.99 (H-2’/5’) together with a singlet at 3.83 for the CH_3_-ester. It is important to note that the three carbonyl-carbons of –COOH, -CONH- and –COOEt or COOMe appeared in the range of 163.0–167.0 ppm together with the ethyl carbons at 61.2 (-COO CH_2_CH_3_), 14.6 (-COOCH_2_CH_3_), and methyl ester-carbon at 52.6 (see Supplementary Figure). Refluxing of **6** and **8** in acetic anhydride for 8 h furnished the corresponding compounds **7** and **9** (Scheme 1). In the cases of **7** and **9**, the two singlets of H-6 and H-3 appeared as one singlet of two equivalent protons at 8.43 and 8.32 ppm, describable for (H-4/7), because of their symmetric structures. This was further documented via the disappearance of the ^13^C-signals of carbonyl-carboxyl and carbonyl-imide and appearance of a dione of two equivalent carbons (C-1/3) instead, observed at about 161.1, and 161.65 ppm confirming a symmetrical dione structure in both **7** and **9** (Figure 2 and Figure 3). 

The IR spectrum of cyclic **7** showed strong absorption peaks at 1688, 1702 and 1780 cm^−1^, consistent with the stretching frequencies of the carbonyl groups (see Supplementary Figure). Treatment of 4,5-dichlorophthalic anhydride with ethylene diamine afforded the corresponding major product **10** and the open anhydride carboxylic acid derivative as a minor product (Scheme 1). The chemical structure of **10** was further confirmed by the above-mentioned spectral analyses. As in the cases of **7** and **9**, compound **10** showed the characteristic features of a 1,3-dione symmetric structure in the form of a singlet ^1^H-resonance at δ 8.22 ppm (H-4/7) and ^13^C-resonance at δ163.0 (C-1/3) for the two equivalent dione carbonyls. Moreover, the presence of an ethylene diamine group was concluded from its two ^1^H-triplets at 3.80 (*J* = 7 Hz, 2H, H-1’) and 2.92 (*J* = 7 Hz, 2H, H-2’) along with their corresponding ^13^C-resonances at 43.3 and 36.5 (-NCH_2_-CH_2_NH_2_). When 4,5-dichlorophthalic anhydride was allowed to react with 2-amino-5-methyl-benzoic acid or 3-amino-2-naphthoic acid in glacial acetic acid under reflux condition, the resulting products **11** and **12** were obtained, and their structures established on the basis of IR, HRMS, and NMR spectral data. Concerning both coupling products (**11**, **12**), they showed more or less the same ^1^H- and ^13^C-resonances as **6** and **8**, except for the interpretation of the resonances corresponding to aminobenzoic or aminonaphthoic moieties instead of the characteristic aminothiophene ethyl or methyl esters. The ^1^H NMR spectrum of **11** displayed a methyl-Ar singlet at δ 2.45 ppm and its ^13^C-resonance at 21.1 ppm together with the carboxyl-carbonyl carbon at 166.5 ppm, while the ^13^C spectrum of **12** showed the carboxyl-carbonyl carbon of the naphthoic acid moiety at 166.6 ppm.

### 2.2. Computational Study

#### 2.2.1. Reactivity Descriptors

For this study, we combined the predicted simulation and practical verification in order to carry out a profound study. We optimized the structures of **1**–**12** via the density functional theory (DFT) method using the Gaussian 09 suite of programs. All compounds were calculated at the B3LYP level with 6–311G(d, p) basis sets. 

In this study, ionization potential (*IP*), electron affinity (*EA*), softness (*S*) chemical potential (*μ*), electrophilicity index (*ω*), and hardness (*η*) were calculated at the same levels; the results are provided in Table 1. From the established theoretical calculations, all chemical descriptors were calculated in order to determine the physical features and chemical reactivity of the target compounds. For instance, compound **5** appeared to have the lowest η value; thus, it can be easily excited. On the other hand, compound **2** had the highest chemical η value; thus, it is the hardest molecule and is more stable. Compound **5** is considered an electron donor, and is good electron acceptor as well due to its lowest IP and highest EA characteristics, respectively. Compound **2** possesses a higher *χ* value than all of the other compounds; thus, it seems to be the best electron acceptor. 

**Figure 4:**
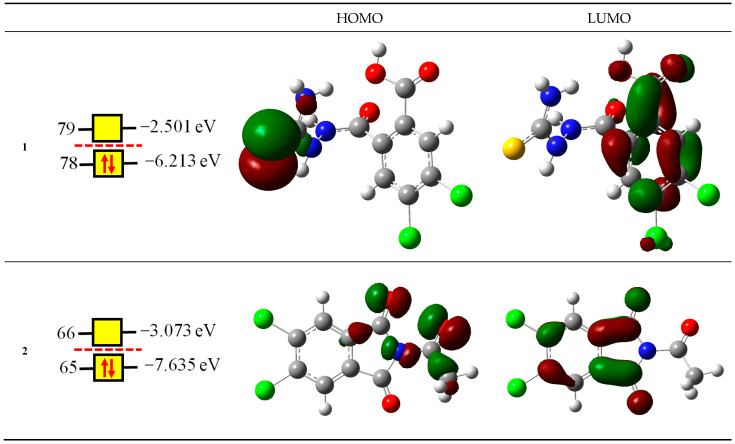

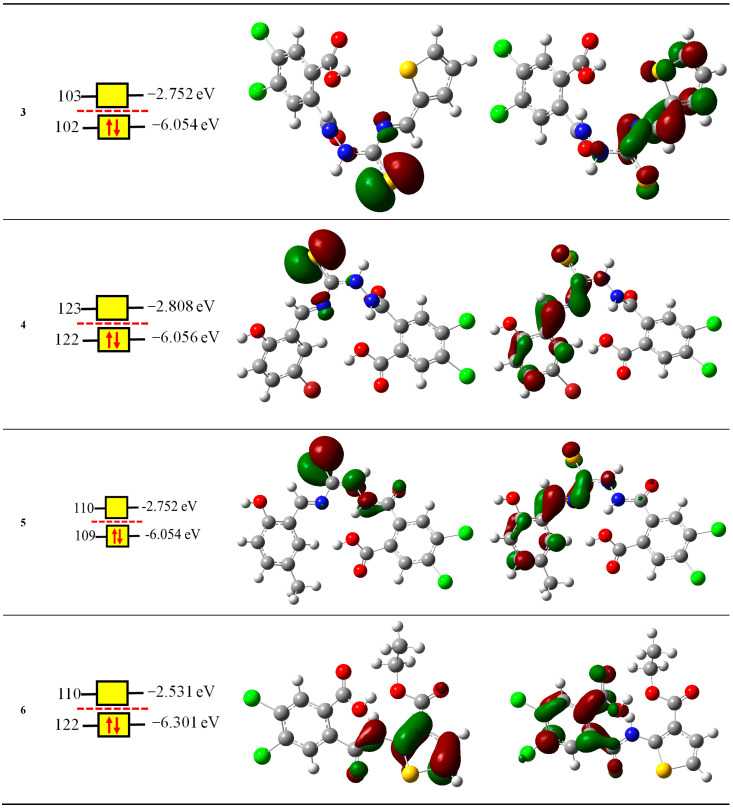

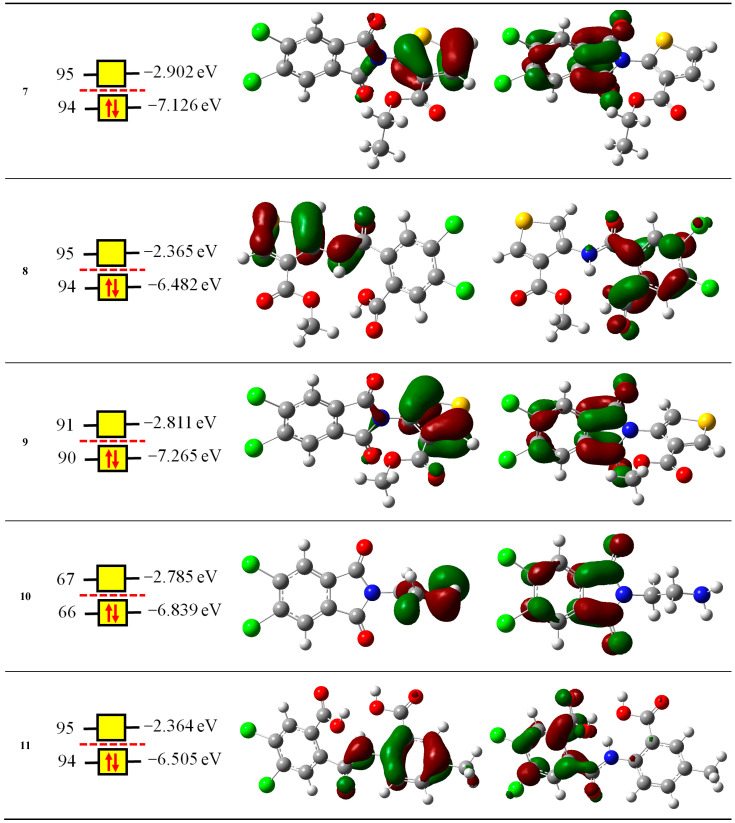

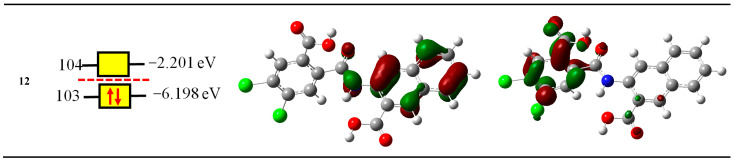
The HOMO and LUMO molecular orbital diagrams of compounds **1**–**12**.

The density distribution of compounds **1**–**5** is mainly on the (Ar−NH) and (NH− CS−NH−NH−) moieties (the electron transfer zones), as shown by their HOMO in Figure 4, while in **6**–**9**, it is mostly distributed on the thiophene moiety. The LUMO spatial distributions of **6**–**12** is located on the carboxylic and benzene, groups (the electron acceptor zones), while they are mostly located on −NH−Ar and (NH− CS−NH−) in **1**–**5**. The CO−N−NH, −N−NH−CS−NH−, Ar−OH, COOH, and Cl-Ar groups thus likely represent the most plausible reaction sites for nucleophilic and electrophilic attacks.

#### 2.2.2. Local Reactivity Descriptors

**Figure 5:**
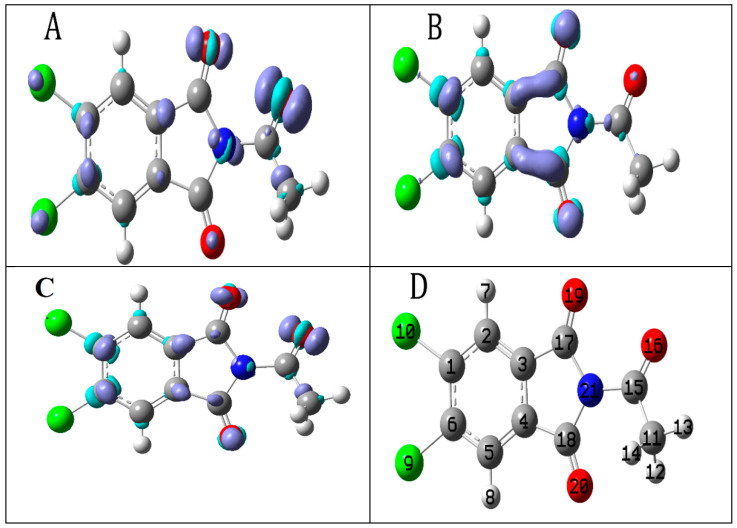
Fukui function describing the sites(lobes) on compound **2** more susceptible to (**A**) electrophilic [*f* ^−^ (r)] attacks, (**B**) nucleophilic [*f* ^+^ (r)] attacks, and (**C**) radical [*f* ^0^ (r)] attacks (**D**) labeled structure.

**Figure 6:**
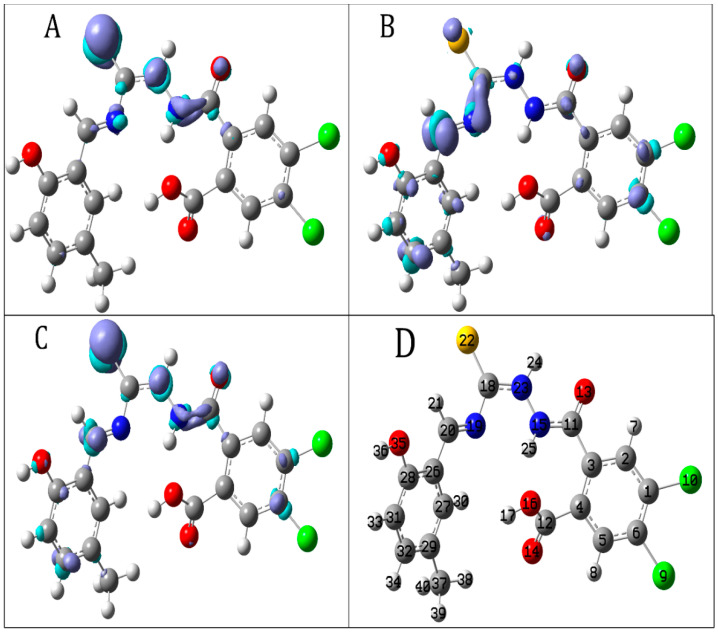
Fukui function describing the sites (lobes) on compound **5** more susceptible to (**A**) electrophilic [*f* ^−^ (r)] attacks, (**B**) nucleophilic [*f* ^+^ (r)] attacks, (**C**) radical [*f* ^0^ (r)] attacks, and (**D**) labeled structure.

Based on the changes in electronic density experienced during the reaction, the calculated Fukui functions were performed in order to determine the molecules’ active sites. The Fukui functions *f*  ^+^ $\begin{array}{c} \to \\ \left(r\right) \end{array}$, *f* ^−^ $\begin{array}{c} \to \\ \left(r\right) \end{array}$ and *f* ^0^ $\begin{array}{c} \to \\ \left(r\right) \end{array}$ can be determined using the following equations:

$$f^{+}=\left[q\left(N+1\right)-q\left(N\right)\right],\;\mathrm{for}\;\mathrm{nucleophilic}\;\mathrm{attack},$$

$$f^{-}=\left[q\left(N\right)-q\left(N-1\right)\right],\;\mathrm{for}\;\mathrm{electrophilic}\;\mathrm{attack},$$

$$f^{0}=\left[q\left(N+1\right)-q\left(N-1\right)\right]/2,\;\mathrm{for}\;\mathrm{radical}\;\mathrm{attack},$$

where *q*(*N*) is the charge on the kth atom for a neutral molecule and *q*(*N* + 1) and *q*(*N* − 1) are the same for its anionic and cationic species, respectively. Using Hirshfeld, the descriptors’ values were calculated at the B3LYP/6-31G (d, p) level. At the DFT level, the most susceptible site to a nucleophilic attack for compound **2** is located on oxygen (O_16_), while for electrophilic attack the most reactive sites are on O_19_ and O_20_. The chloro atoms Cl_9_ and Cl_10_ are the most reactive sites for a free radical attack (Figure 5). In the case of **5**, the C_20_ is the most susceptible site to nucleophilic attacks, while S_22_ is susceptible to electrophilic and free radical attack (Figure 6). The Fukui data are presented in Tables S1 and S2.

#### 2.2.3. Molecular Electrostatic Potential (MEP)

**Figure 7:**
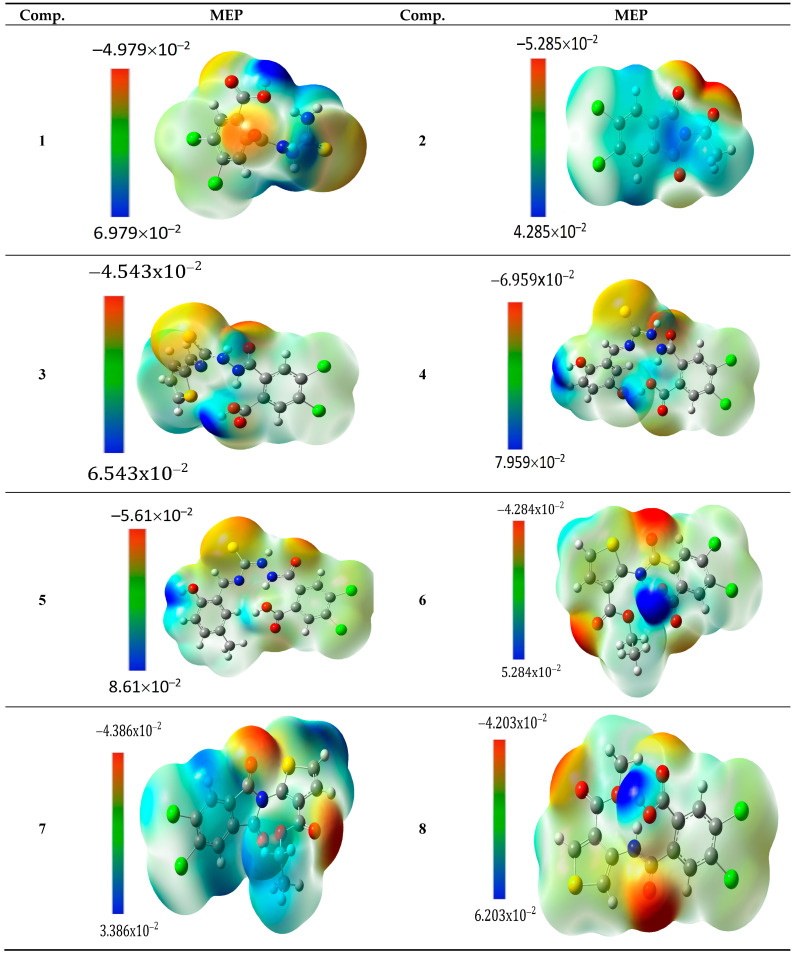

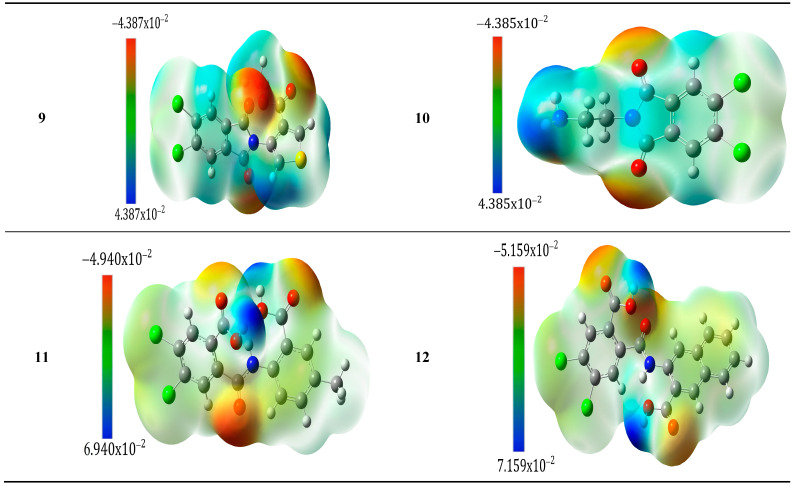
Molecular electrostatic potential of compounds **1**–**12**.

The various electrostatic potential values at the surface are represented by different colors: red for electronegative, blue for positive electrostatic potential, and green for zero potential (Figure 7). The increase in electrostatic potential is ordered as red < orange < yellow < green < blue. The MEP was generated at DFT/B3LYP/6-311G(d, p) levels of theory. Figure 7 shows how the MEP electronic density can help to find places where electrophilic and nucleophilic attacks can happen and where hydrogen bonds can form. The negative areas (red color) of MEP are related to electrophilic reactivity and the positive areas (blue color) to nucleophilic reactivity. According to the MEP maps (Figure 7), the negative region of compound **2** is mainly focused on the oxygen in the C=O group (more color intensity), therefore, this is a useful region for nucleophilic activity. In addition, the lowest electron density was found in compound **5** at 8.610 × 10^−2^ (a.u.) with the highest intensity blue colour shown for the hydrogen atom in O-H, making it an appropriate site for electrophilic attraction.

#### 2.2.4. Vibrational Analysis

From the experimental and calculated FT–IR spectra (see Supplementary Figures), a slight difference observed between the practical and the theoretical values could be attributed to the calculations being prepared for a free molecule in vacuum, while practically this is carried out with solid samples. The harmonic frequencies were calculated by the B3LYP method using 6–311G(d, p) basis sets, then scaled by 0.96050. In this study, compound **2** was selected; its experimentally detected and theoretically determined harmonic vibrational frequencies and its correlations are presented in Table 2. The vibrational band assignments were made using the PED analysis and the animation option in the Gauss View 5.0 graphical interface for Gaussian. The maximum number of values determined by the B3LYP/6–311G(d, p) method was in fairly good agreement with the experimental values, and was further confirmed by the scale factors used to obtain the scaled frequencies.

#### 2.2.5. NMR Spectroscopy

**Figure 8:**
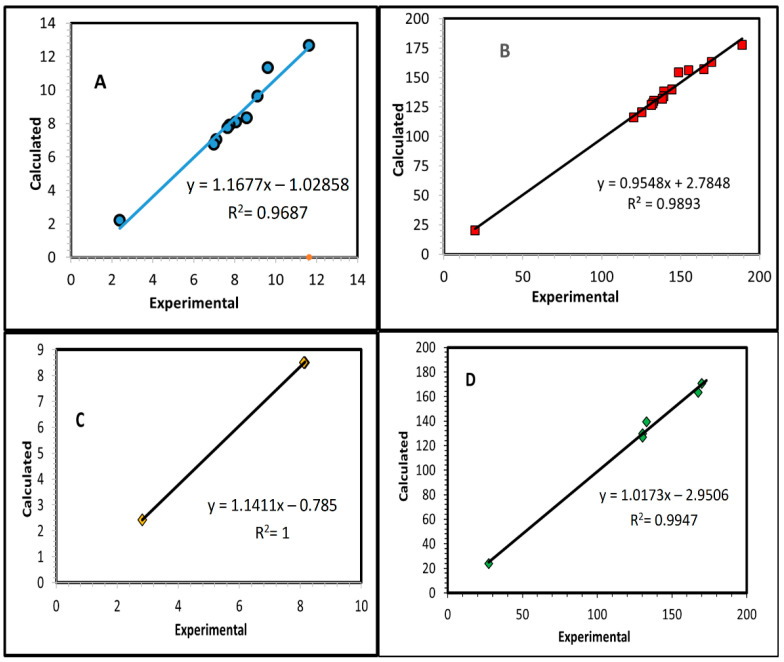
Correlation graphics of calculated and experimental chemical shifts of ^1^H and ^13^C of compound **5** in (**A**,**B**), respectively, as well as ^1^H and ^13^C of compound **2** in (**C**,**D**), respectively.

The ^1^H NMR and ^13^C NMR spectra of the synthesized compounds are shown in the supplementary materials. This assignment is further supported by DFT/B3LYP calculations in DMSO using the basis sets 6-311G(d, p) with the GIAO solvation method (IEF-PCM). The practical and predicted chemical shift values are presented in Tables S3 and S4. Moreover, the correlations of the experimental and calculated ^1^H and ^13^C chemical shifts are shown in Figure 8. The ^1^H and ^13^C NMR chemical shift correlation coefficients (R2) were found to be 0.978203291 and 0.97954627 (6-311G(d, p)) for compounds **2** and **5**, respectively. The observed correlation coefficient (R2) value shows that the practical and calculated chemical shift values were very close to each other [21,22].

## 3. Conclusions

The target compounds **1**–**12** were successfully synthesized and their structures established on the basis of their NMR, IR, and MS spectral data. Practical and theoretical vibrational analysis of compound **2** was performed and showed fairly good agreement. The experimental data were correlated with DFT theoretical calculations employing (DFT/B3LYP) with the 6-311G(d, p) basis sets. Different electronic and reactivity descriptors of the target compounds **1**–**12** were calculated in order to clarify their physical and chemical features. Various 4,5-dichlorophthalic anhydride derivatives were submitted for biological evaluation and will be discussed in a full study in forthcoming research. 

## 4. Materials and Methods

### 4.1. General Information

NMR spectra were measured in DMSO-*d*_6_ on a Bruker AMX 700 spectrometer operated at 700 MHz for ^1^H NMR and 175 MHz for ^13^C NMR. High-resolution mass spectra (EI-HRMS) were measured on a JEOL MStation JMS-700 system. A Brucker Alpha II FTIR-ATR spectrometer was used to record the IR spectra (KBr, v, cm^−1^). Melting points (uncorrected) were measured using a STUART SMP 10 melting point apparatus. Monitoring the reactions and checking compound purity were carried out by TLC on a DC Mikrokarten polygram SIL G/UV254 (Macherey-Nagel Firm, Duren) with a thickness of 0.25 mm.

#### 4.1.1. 2-(2-Carbamothioylhydrazine-1-carbonyl)-4,5-dichlorobenzoic Acid (1)

4,5-Dichlorophthalic anhydride (1 mmol) was dissolved in boiling glacial acetic acid (15 mL), then thiosemicarbazide (1.5 mmol) was added and the reaction mixture was refluxed for 6 hrs. The obtained solid was filtrated and washed with water and ether. Yield (75%); mp 220 °C; ^1^H NMR (700 MHz, DMSO-*d_6_*): *δ* 14.11 (br s, 1H, −COOH), 10.48 (s, 1H, −CO.NH.NH.CS−), 9.47 (s, 1H, −CO.NH.NH.CS−), 8.22 (s, 1H, H-6), 8.07 (s, 1H, H-3), 7.85, 7.43 (each s, each 1H,–NH2); ^13^C NMR (175 MHz, DMSO-*d_6_*): *δ* 182.7 (C=S),166.5 (C-7), 166.0 (−CONH−), 136.8 (C-4), 135.4 (C-5), 133.3 (C-2), 131.6 (C-1), 130.9 (C-6),130.7 (C-3); IR (KBr, v, cm-^1^): 1663 and 1702 cm^−1^ (C=O), 3243–3439 cm^−1^ (NH &NH_2_); HRMS (EI), *m*/*z* calcd. for C_9_H_7_Cl_2_N_3_O_3_S (M)^•+^ 306.9556, found 306.9503.

#### 4.1.2. 2-Acetyl-5,6-dichloroisoindoline-1,3-dione (2)

Compound **1** (1 mmol) was refluxed with acetic anhydride (10 mL) for 7 hrs. The reaction mixture was cooled and the resulting solid was collected. Yield (60%); mp 190 °C; ^1^H NMR (700 MHz, DMSO-*d_6_*): *δ* 8.50 (s, 2H, H-4/7), 2.43 (s, 3H, −NCOCH_3_); ^13^C NMR (175 MHz, DMSO-*d_6_*): *δ* 170.0 *(*(−NCOCH3), 163.3 (C-1/3), 139.5 (C-5/6), 129.6 (C-3a,3b), 127.2 (C-4/7), 24.7 (−NCOCH_3_); IR (KBr, v, cm^−1^): 1727 and 1796 cm^−1^ (C=O), HRMS (EI), *m*/*z* calcd. for C_10_H_5_Cl_2_NO_3_ (M)^•+^ 256.9646, found 256.9598.

#### 4.1.3. General Procedure for Synthesis of (3−5)

A mixture of aldehyde (1.1 mmol) and compound **1** (1.1 mmol) was refluxed in DMF (15 mL) in the presence of few drops of glacial acetic acid for 5–9 hrs. After cooling, the precipitate was filtered off, washed with water, and dried to obtain the pure compounds **3–5.**

##### (*E*)-4,5-Dichloro-2-(2-((thiophen-2ylmethylene)carbamothioyl)hydrazine-1-carbonyl) benzoic Acid (**3**)

Yield (43%); mp 187 °C; ^1^H NMR (700 MHz, DMSO-*d_6_*): *δ* 11.42 (s, 1H, −CONH. NH.CS−), 10.24 (s, 1H, −CO.NH.NH.CS−), 8.30 (s, 1H, −N=CH-6’), 8.21 (s, 1H, H-6), 8.17 (br s, 1H, H-5’), 8.15 (s, 1H, H-3), 7.33 (dd, J = 7.0,1.5 Hz, 1H, H-4’), 6.82 (d, J = 7.0 Hz, 1H, H-3’); ^13^C NMR (175 MHz, DMSO-*d_6_*): *δ* 178.3 (C=S), 166.4 (C-7), 166.0 (−CONH−), 155.7 (−N=CH−), 149.8 C-2’), 137.8 (C-4), 135.3 (C-5), 133.5 (C-2,1),130.8 (C-6), 128.8 (C-3), 123.4 (C-3’), 118.5 (C-5’), 111.6 (C-4’); IR (KBr, v, cm^−1^): 1693 cm^−1^ (C=O), 3405 cm^−1^ (-NH-); HRMS (EI), *m*/*z* calcd. for C_14_H_9_Cl_2_N_3_O_3_S_2_ (M)^•+^ 400.9462, found 400.9411.

##### (*E*)-2-(2-((5-Bromo-2-hydroxybenzylidene) carbamothioyl)hydrazine-1-carb onyl)-4,5-dichlorobenzoic Acid (**4**)

Yield (47%); mp 203 °C; ^1^H NMR (700 MHz, DMSO-*d_6_*): *δ* 11.42 (s, 1H, −CONH. NH.CS−), 10.98 (s, 1H, -OH-2’), 10.20 (s, 1H, −CO.NH.NH.CS−), 8.33 (s, 1H, −N=CH-7’), 8.21 (s, 1H, H-6), 8.16 (s, 1H, H-3), 7.72 (d, J = 1.7 Hz, 1H, H-6’), 7.33 (dd, J = 7.5,1.7 Hz, 1H, H-4’), 6.81 (d, J = 7.5 Hz, 1H, H-3’); ^13^C NMR (175 MHz, DMSO-*d_6_*): *δ* 179.0 (C=S), 166.4 (C-7), 166.0 (-CONH-), 160.0 (C-2’), 156.0 (−N=CH-7’), 138.9 (C-4’), 137.5 (C-4), 134.5 (C-5), 133.3 (C-2/1), 131.1 (C-6/6’), 128.0 (C-3), 120.4 (C-1’), 118.0 (C-3’), 111.4 (C-5’); IR (KBr, v, cm^−1^): 1689 cm^−1^ (C=O), 3367cm^−1^ (-NH-); HRMS (EI), *m*/*z* calcd. for C_16_H_10_BrCl_2_N_3_O_4_S (M)^•+^ 488.8952, found 488.9001.

##### (*E*)-4,5-Dichloro-2-(2-((2-hydroxy-5-methylbenzylidene)carbamothioyl)hydrazine-1-carbonyl)benzoic Acid (**5**)

Yield (45%); mp 239 °C; ^1^H NMR (700 MHz, DMSO-*d_6_*): *δ* 11.35 (s, 1H, −CONH. NH.CS−), 9.63 (s, 1H, −CO.NH.NH.CS−), 8.34 (s, 1H, −N=CH-7’), 8.11 (s, 1H, H-6), 7.92 (s, 1H, H-3), 7.75 (br s, 1H, H-6’), 7.03 (br d, J = 7.5 Hz, 1H, H-4’), 6.76 (d, J = 8.0 Hz, 1H, H-3’), 2.22 (s, 3H, Ar-CH3); ^13^C NMR (175 MHz, DMSO-*d_6_*): *δ* 178.1 (C=S), 166.4 (C-7), 166.0 (−CONH−), 162.7 (C-2’), 154.6 (−N=CH-7’), 140.3 (C-4’), 137.6 (C-4), 134.5 (C-5), 133.3 (C-2/4’/1), 132.1 (C-6/5’), 128.8 (C-3), 127.1 (C-6’), 120.6 (C-1’), 116.3 (C-3’), 20.45 (Ar-CH3); IR (KBr, v, cm^−1^): 1703 cm^−1^ (C=O), 3400 cm^−1^ (-NH-); HRMS (EI), *m*/*z* calcd. for C_17_H_13_Cl_2_N_3_O_4_S (M)^•+^ 425.0003, found 425.0045.

#### 4.1.4. General Procedure for Preparation of (6−9)

A mixture of 4,5-dichloro-phthalic anhydride (1 mmol) and 2-amino-thiophene-3-carboxylic acid ethyl ester (1.3 mmol) or methyl-4-minothiophene-3-carboxylate hydrochloride (1.3 mmol) was stirred under reflux conditions in glacial acetic acid (15 mL) or DMF (15 mL) for 7 h. After cooling, the solid was filtrated and washed with water to produce compounds **6** and **8** as the final products. Compounds **6** or **8** (1 mmol) was refluxed in acetic anhydride (10 mL) for 8 h. After cooling, the obtained solid compound (**7** or **9**) was collected and dried (the reaction for preparation **8** proceeded in trimethylamine and used DMF as solvent). 

##### 4,5-Dichloro-2-((3-(ethoxycarbonyl)thiophen-2-yl)carbamoyl)benzoic Acid (**6**)

Yield (76%); mp 170 °C; ^1^H NMR (700 MHz, DMSO-*d6*): *δ* 11.22 (1H, s, COOH), 8.38 (s, 1H, −NH), 8.09 (s, 1H, H-6), 8.04 (s, 1H, H-3), 7.23 (br s, 1H, H-5’), 7.13 (br s, 1H, H-4’), 4.29 (q, J = 7 Hz, 2H, −CH_2_CH_3_), 1.29 (t, J = 7 Hz, 3H, −CH_2_CH_3_); ^13^C NMR (175 MHz, DMSO-*d6*): *δ* 167.0 (C-2’), 166.0 (C-7), 165.7 (−CONH−), 164.6 (−COOCH_2_CH_3_), 138.5 (C-4), 136.5 (C-5), 133.2 (C-2), 132.2 (C-1), 130.6 (C-6/3), 124.3 (C-4’), 117.3 (C-5’), 114.3 (C-3’), 61.2 (−COOCH_2_CH_3_), 14.6 (−COOCH_2_CH_3_); IR (KBr, v, cm^−1^):1730, 1662 cm^−1^ (C=O), 3270, 3376 cm^−1^ (-NH-); HRMS (EI), *m*/*z* calcd. for C_15_H_11_Cl_2_NO_5_S (M)^•+^ 386.9735, found 386.9694.

##### Ethyl 2-(5,6-dichloro-1,3-dioxoisoindolin-2-yl)thiophene-3-carboxylate (**7**)

Yield (52%); mp 140 °C; ^1^H NMR (700 MHz, DMSO-*d_6_*): *δ* 8.43 (s, 2H, H-4/7), 7.79 (br s, 1H, H-5’), 7.51 (br s, 1H, H-4’), 4.10 (q, J = 7 Hz, 2H, -CH_2_CH_3_), 1.02 (t, J = 7 Hz, 3H, −CH_2_CH_3_); ^13^C NMR (175 MHz, DMSO-*d_6_*): *δ*164.9 (−COOCH_2_CH_3_), 161.1 (C-1/3), 139.5 (C-5/6), 136.8 (C-2’), 131.7 (C-4’), 129.8 (C-3a,3b), 127.7 (C-5’), 127.6 (C-3’), 126.3 (C-4/7), 61.1 (−COOCH_2_CH_3_), 13.9 (−COOCH_2_CH_3_); IR (KBr, v, cm^−1^): 1688, 1702 and 1780 cm^−1^ (C=O); HRMS (EI), *m*/*z* calcd. for C_15_H_9_Cl_2_NO_4_S (M)^•+^ 368.9628, found 368.9674.

##### 4,5-Dichloro-2-((4-(methoxycarbonyl)thiophen-3-yl)carbamoyl) benzoic Acid (**8**)

Yield (63%); mp 195 °C; ^1^H NMR (700 MHz, DMSO-*d_6_*): *δ* 10.25 (1H, s, −COOH), 8.41 (s, 1H, −NH), 8.06 (s, 1H, H-6), 8.00 (s, 1H, H-3), 7.99 (m, 2H, H-2’/5’), 3.83 (s, 3H, −COOCH_3_); ^13^C NMR (175 MHz, DMSO-*d_6_*): *δ* 166.0 (C-7), 164.1 (−CONH−), 162.8 (−COOCH_3_), 137.7 (C-4), 135.7 (C-5), 135.0 (C-4’), 133.0 (C-2), 132.0 (C-2’), 131.6 (C-1), 130.1 (C-6/3), 122.2 (C-3’),112.9 (C-5’), 114.3 (C-3’), 52.6 (−COOCH_3_); IR (KBr, v, cm^−1^):1723, 1656 cm^−1^ (C=O), 3282, 3400cm^−1^ (-NH-); HRMS (EI), *m*/*z* calcd. for C_14_H_9_Cl_2_NO_5_S (M)^•+^ 372.9578, found 372.9612.

##### Methyl 4-(5,6-dichloro-1,3-dioxoisoindolin-2-yl)thiophene-3-carboxylate (**9**)

Yield (52%); mp 165 °C; ^1^H NMR (700 MHz, DMSO-*d_6_*): δ 8.54 (d, J = 7 Hz, 1H, H-2’), 8.32 (s, 2H, H-4/7), 7.90 (d, J = 7 Hz, 1H, H-5’), 3.67 (s, 3H, −OCH_3_); ^13^C NMR (175 MHz, DMSO-*d_6_*): δ 165.5 (−COOCH_3_), 161.9 (C-1/3), 138.7 (C-5/6), 135.7 (C-4’), 132.2 (C-2’), 129.6 (C-3a,3b), 127.9 (C-3’), 126.4 (C-4/7,5’), 52.5 (−COOCH_3_); HRMS (EI), *m*/*z* calcd. for C_14_H_7_Cl_2_NO_4_S (M)^•+^ 354.9473, found 354.9433.

##### 2-(2-aminoethyl)-5,6-dichloroisoi ndoline-1,3-dione (**10**)

A mixture of 4,5-dichlorophthalic anhydride (1 mmol) and ethylene diamine (4 mmol) was stirred under reflux in glacial acetic acid (10 mL) for 8 hrs. After cooling, the precipitate was filtrated and washed with water to give the final product. Yield (65%); mp 231 °C; ^1^H NMR (700 MHz, DMSO-*d_6_*): *δ* 8.22 (s, 2H, H-4/7), 7.95, 7.73 (each s, each 1H,–NH_2_), 3.80 (t, J = 7 Hz, 2H, H-1’), 2.92 (t, J = 7 Hz, 2H, H-2’); ^13^C NMR (175 MHz, DMSO-*d_6_*): *δ* 163.0 (C-1/3), 137.8 (C-5/6), 126.0 (C-3a,3b), 125.7 (C-4/7), 43.3, 36.5 (-NCH_2_-CH_2_NH_2_); HRMS (EI), *m*/*z* calcd. for C_10_H_8_Cl_2_N_2_O_2_ (M)^•+^ 257.9963, found 258.0001.

##### 2-((2-Aminoethyl)carbamoyl)-4,5-dichlorobenzoic Acid (10-minor)

^1^H NMR (700 MHz, DMSO-*d_6_*): *δ* 8.70 *(s*, 1H, -NH), 8.21 (s, 1H, H-6), 8.06 (s, 1H, H-3), 7.75, 7.55 (each s, each 1H,–NH_2_), 3.73 (t, J = 7 Hz, 2H, H-1’), 2.74 (t, J = 7 Hz, 2H, H-2’); ^13^C NMR (175 MHz, DMSO-*d_6_*): *δ* 166.7 (C-7), 166.5 (-CONH-),137.1 (C-4), 134.3 (C-5), 133.2 (C-2), 130.9 (C-1), 130.8 (C-6), 129.6 (C-3), 31.0 (-NHCH_2_-CH_2_NH_2_).

#### 4.1.5. General Procedure for Preparation of 11 and 12

A mixture of 4,5-dichlorophthalic anhydride (1 mmol) and 2-amino-5-methyl-benzoic acid (1.2 mmol) or 3-amino-2-naphthaoic acid (1.2 mmol) was refluxed in glacial acetic acid for 6–8 hrs. After cooling, the obtained solid was collected and washed with water to obtain the final products.

##### 2-((2-Carboxy-4-methylphenyl)carbamoyl)-4,5-dichlorobenzoic Acid (**11**)

Yield (66%); mp 234 °C; ^1^H NMR (700 MHz, DMSO-*d_6_*): *δ* 14.15, 13.35 (each br s, each 1H, 2x –COOH), 8.33 (s, 1H, −CO.NH−), 8.06 (s, 1H, H-6), 8.01 (s, 1H, H-3), 7.89 (br s, 1H, H-3’), 7.59 (br d, J = 7.5 Hz, 1H, H-5’), 7.42 (d, J = 8.0 Hz, 1H, H-6’), 2.45 (s, 3H, Ar-CH_3_); ^13^C NMR (175 MHz, DMSO-*d_6_*): *δ* 166.5 (C-7’), 166.2 (C-7), 165.9 (-CONH-), 140.0 (C-1’), 138.3 (C-4), 135.2 (C-5), 134.1 (C-2), 132.1 (C-1/5’), 131.6 (C-3’), 130.8 (C-6), 130.0 (C-3), 129.0 (C-4’), 126.2 (C-2’/6’), 21.1 (Ar-CH_3_); HRMS (EI), *m*/*z* calcd. for C_16_H_11_Cl_2_NO_5_ (M)^•+^ 367.0014, found 367.0059.

##### 3-(2-Carboxy-4,5-dichlorobenzamido)-2-naphthoic Acid (**12**)

Yield (56%); mp 217 °C; ^1^H NMR (700 MHz, DMSO-*d_6_*): *δ* 13.25 (br s, 2H, 2x –COOH), 8.74 (s, 1H, H-9’), 8.37 (s, 1H, −CO.NH−), 8.06 (s, 1H, H-6), 8.01 (s, 1H, H-3), 8.24 (br d, J = 7.5 Hz, 1H, H-8’), 8.12 (s, 1H, H-4’), 8.09 (br d, J = 7.5 Hz, 1H, H-5’), 7.77 (br t, J = 7.5 Hz, 1H, H-6’), 7.74 (br t, J = 7.5 Hz, 1H, H-7’); ^13^C NMR (175 MHz, DMSO-*d_6_*): *δ* 166.6 (C-7’), 166.2 (C-7/-CONH-), 138.4 (C-4/3’), 134.7 (C-5), 133.3 (C-2), 132.4 (C-4’a), 132.1 (C-1/1’), 130.6 (C-6), 129.8 (C-3), 129.6 (C-8’), 128.6 (C-6’), 128.2 (C-8’a), 127.7 (C-5’), 126.3 (C-2’/4’/7’); HRMS (EI), *m*/*z* calcd. for C_19_H_11_Cl_2_NO_5_ (M)^•+^ 403.0014, found 403.0048.

### 4.2. Computational Methods

Optimization of the molecular structures of compounds **1**–**12** in the ground state was performed by density functional theory (DFT/B3LYP) using the 6-311G(d, p) basis [23,24]. The vibrational frequencies, optimized geometrical parameters, and energy were calculated by employing the GAUSSIAN 09 W package [25]. The Gauss View 6.0 program was utilized to build MEP, HOMO, and LUMO energy distributions and to optimize the molecular geometry [26]. Calculation of the potential energy distribution (PED) was carried out with the assistance of the VEDA 4 software package [27]. The symmetry analysis of several compounds is presented in detail in order to describe the basis behind the assignments and improve the agreement between the predicted and observed results [28,29,30,31,32,33,34]. Fukui function analysis was performed in the program Multiwfn 43, using the corresponding monodeterminant wave functions of the selected structures [35].

## Figures and Tables

**Table 1:** Calculated number of occupied molecular orbitals (NOMO), *IP, EA*, *μ*, *χ*, *η*, *S*, *ω*, and index *N* for **1**–**12** at the B3LYP/6-311 G (d, p) level.

Compounds	NOMO	*IP* eV	*EA* eV	*χ* eV	*μ* eV	*η* eV	*S* eV^−1^	*ω* eV	*N* eV
**1**	78	8.3026	0.8264	4.5645	−4.5645	7.4761	0.1338	1.3934	2.9079
**2**	65	9.3286	1.3546	5.3416	−5.3416	7.9740	0.1254	1.7891	1.4862
**3**	102	7.8832	1.3558	4.6195	−4.6195	6.5274	0.1532	1.6346	3.0676
**4**	122	7.8169	1.4795	4.6482	−4.6482	6.3373	0.1578	1.7046	3.0646
**5**	109	7.5565	1.5188	4.5376	−4.5376	6.0377	0.1656	1.7051	3.1522
**6**	99	7.9733	1.0535	4.5134	−4.5134	6.9198	0.1445	1.4719	2.82
**7**	94	8.7139	1.2813	4.9976	−4.9976	7.4326	0.1345	1.6801	1.9955
**8**	95	8.1561	0.8204	4.4882	−4.4882	7.3357	0.1363	1.373	2.6394
**9**	90	8.7966	1.1712	4.9839	−4.9839	7.6254	0.1311	1.6287	1.8566
**10**	66	8.7233	1.0662	4.8947	−4.8947	7.657	0.1306	1.5645	2.2821
**11**	94	8.094	0.95	4.522	−4.522	7.144	1.4312	2.6159	8.094
**12**	103	7.7296	0.9403	4.335	−4.335	6.7893	0.1473	1.3839	2.9233

**Table 2:** The calculated and experimental wavenumber (cm^−1^) B3LYP/6–311G(d, p) of compound **2**.

Calculated Wavenumber (cm^−1^) B3LYP/6–311G(d, p)		Experimental	Vibrational Band Assignment (% PED)
Un scaled	Scaled	υ(IR)	
470	451	434.98	(64) τHCCC
579	556	521.94	(10) βCCC+CCO
615	591	587.74	(59) βCCCl+CCO+CNC
632	607	617.25	(30) βCCC
766	736	733.31	(29) βCCC
794	762	770.25	(10) βOCC
875	840	802.19	(43) βCCC+CCCl+CCO
932	895	896.47	(13) βHCH
964	926	977.47	(66) βHCH
1105	1061	1041.79	(11) βHCC
1148	1102	1096.95	(12) βCCCl+CCC+CCO
1278	1228	1211.72	(16) ʋNC
1334	1281	1256.44	(35) ʋCC+NC
1395	1340	1309.31	(54) ʋCC+NC
1463	1405	1373.9	(44) ʋC=C+NC
1483	1424	1427.28	(70) ʋCC
1634	1569	1606.07	(11) ʋCC
1814	1742	1727.62	(76) ʋOC
1870	1796	1796.96	(76) ʋOC
3060	2938	2934.53	(95) ʋCH_3_

## Supplementary Materials

The following are available online at https://www.mdpi.com/article/10.3390/molecules27113550/s1, supporting IR spectra and supporting NMR spectra for the target compounds, supporting EI-HRMS spectra for compounds **6** and **7**, Tables S1 and S2: Values of the Fukui function of the compounds **2** and **5**, Tables S3 and S4: Experimental and calculated ^13^C& ^1^H isotropic chemical shifts (ppm) for compounds **2** and **5**.

## Sample Availability

Samples of the compounds **1**–**12** are available from the authors.
