# Supplementary material for: Reactivity of 4,5-Dichlorophthalic Anhydride towards Thiosemicarbazide and Amines: Synthesis, Spectroscopic Analysis, and DFT Study
Source: Molecules. 2022 May 31;27(11):3550. doi: 10.3390/molecules27113550 (PMC9182083; doi:10.3390/molecules27113550)
Supplement: Supplementary file 1 [file molecules-27-03550-s001.zip › molecules-1730036-supplementary.pdf]

Supporting IR spectra

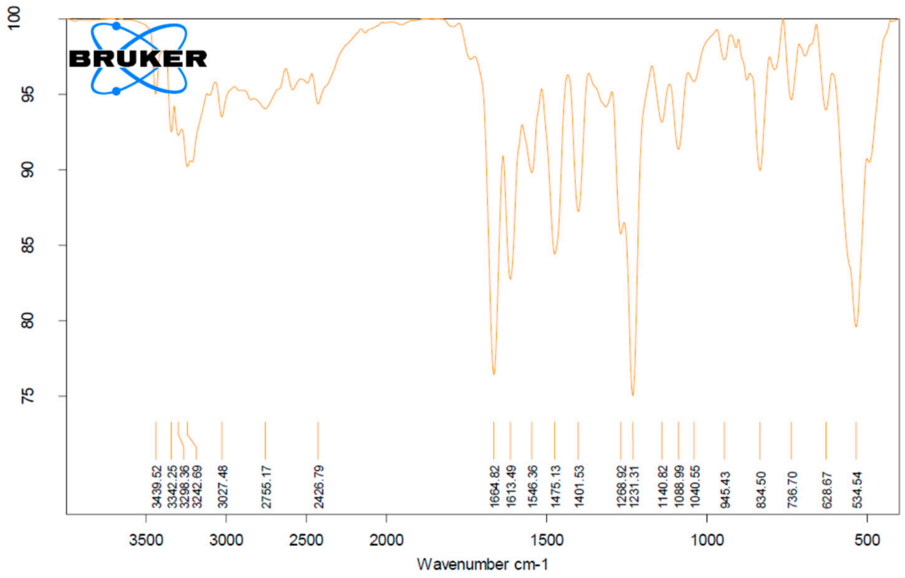

Experimental IR spectrum of compound 1

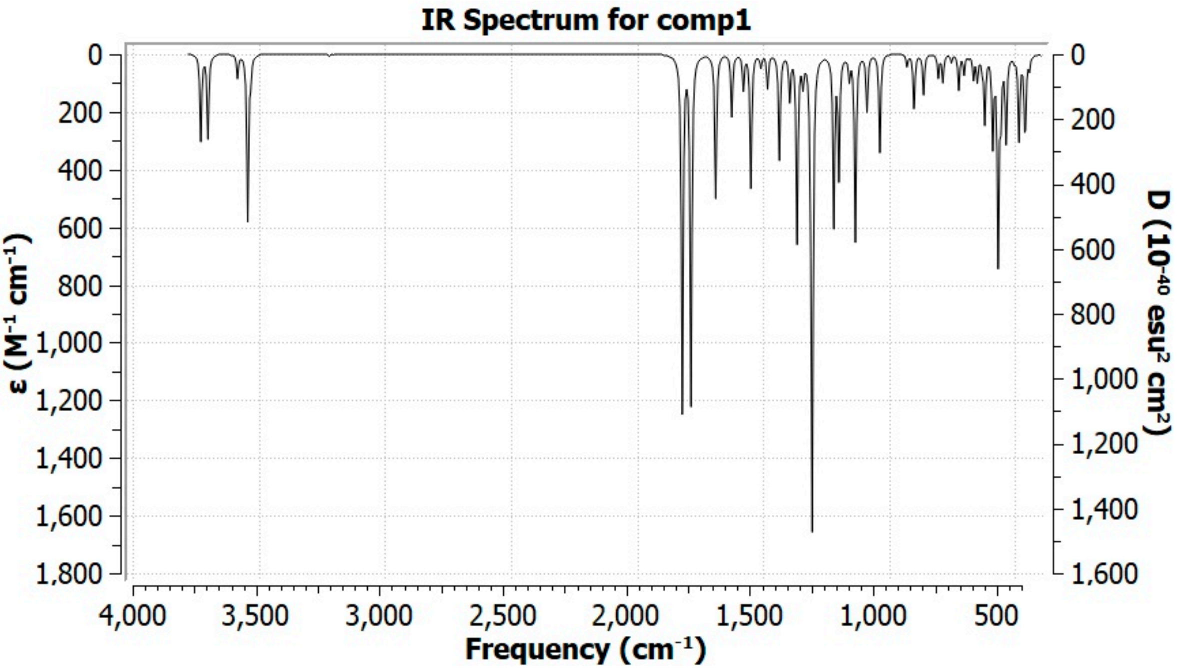

Predicted IR spectrum of compound 1

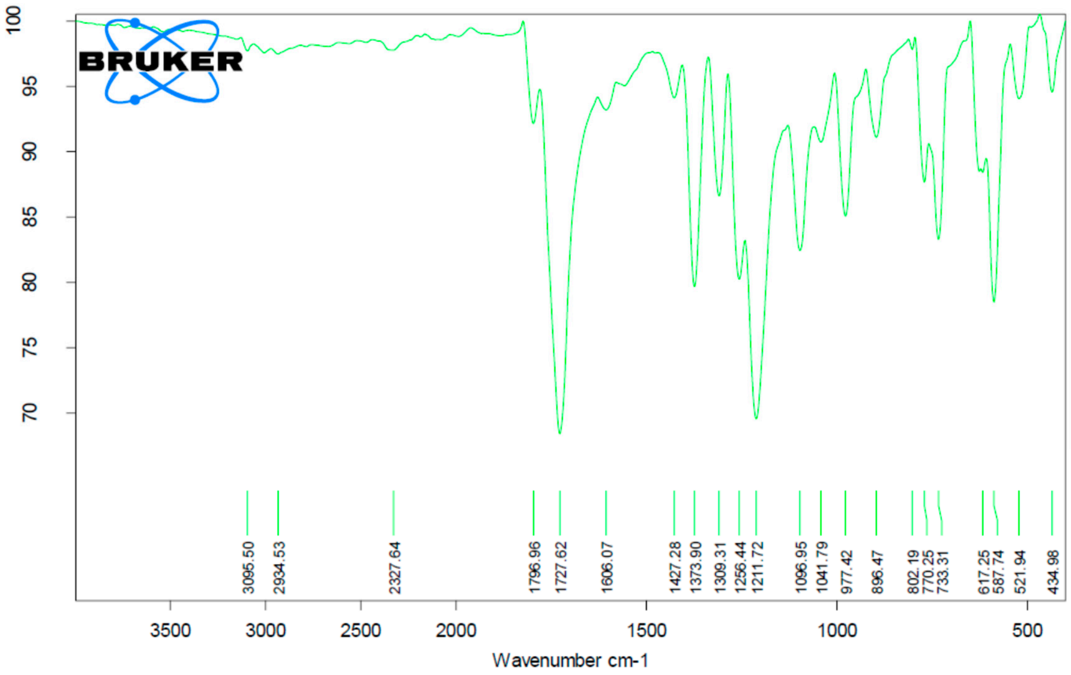

Experimental IR spectrum of compound 2

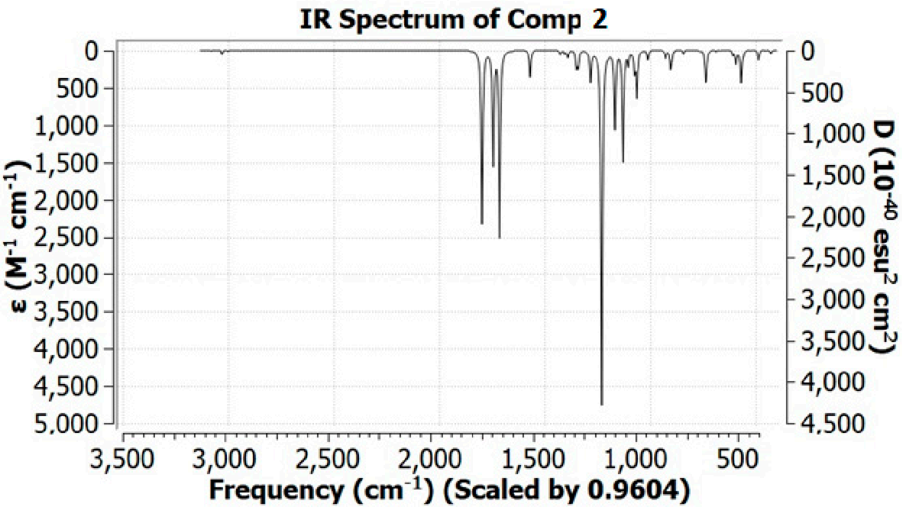

Predicted IR spectrum of compound 2

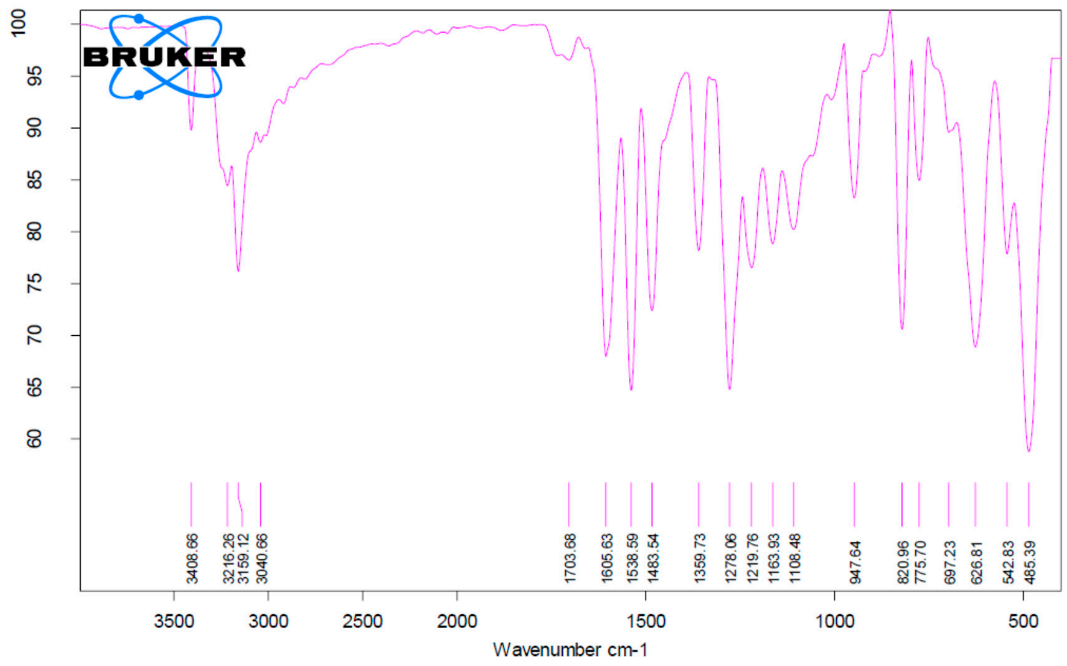

Experimental IR spectrum of compound 5

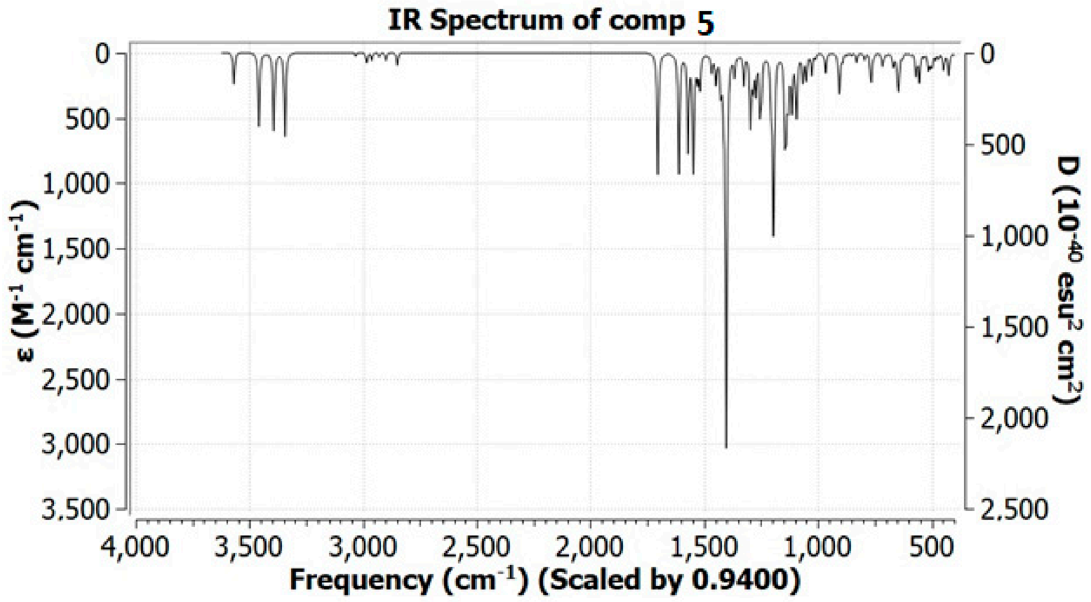

Predicted IR spectrum of compound 5

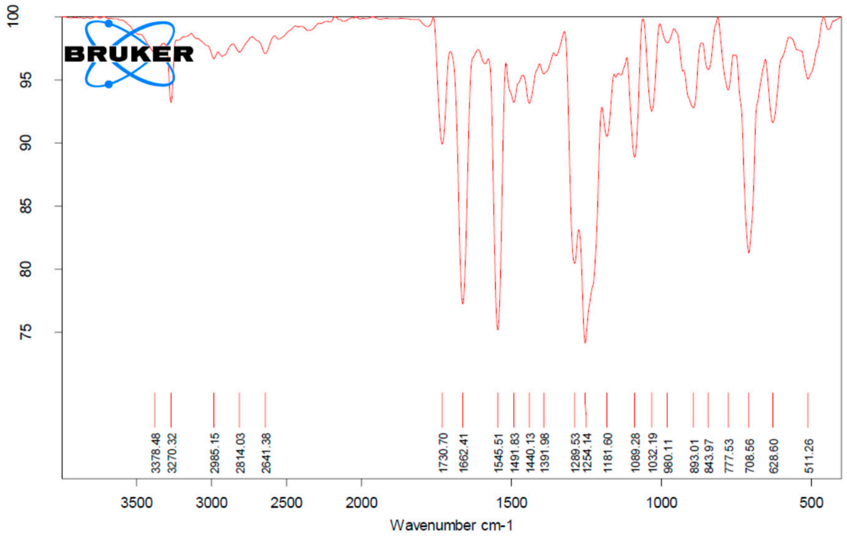

Experimental IR spectrum of compound 6

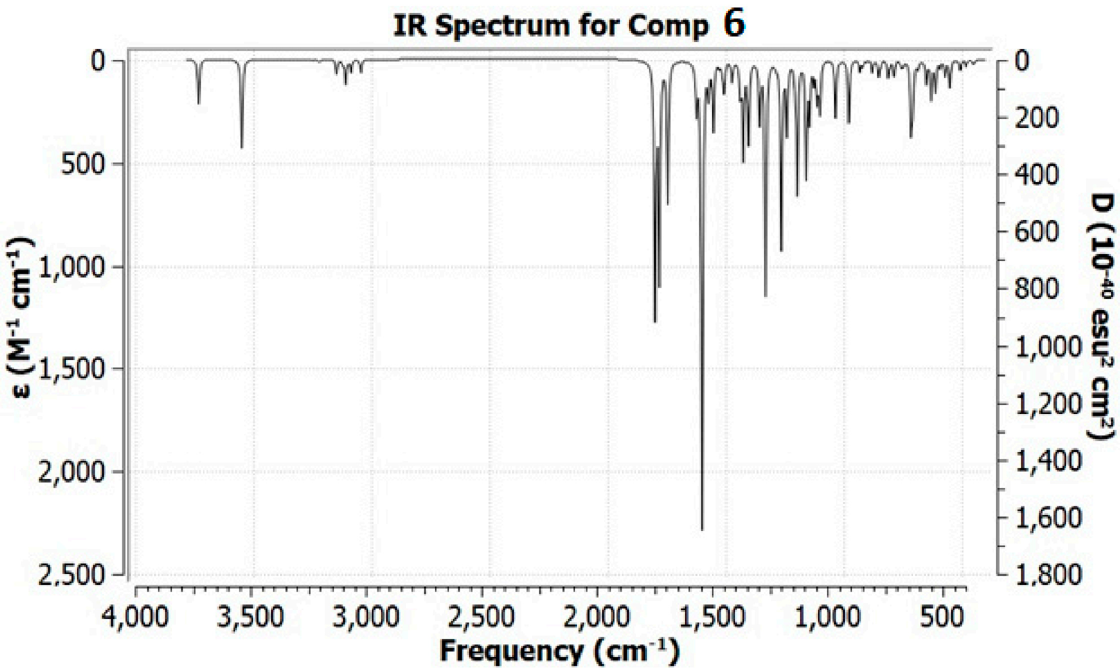

Predicted IR spectrum of compound 6

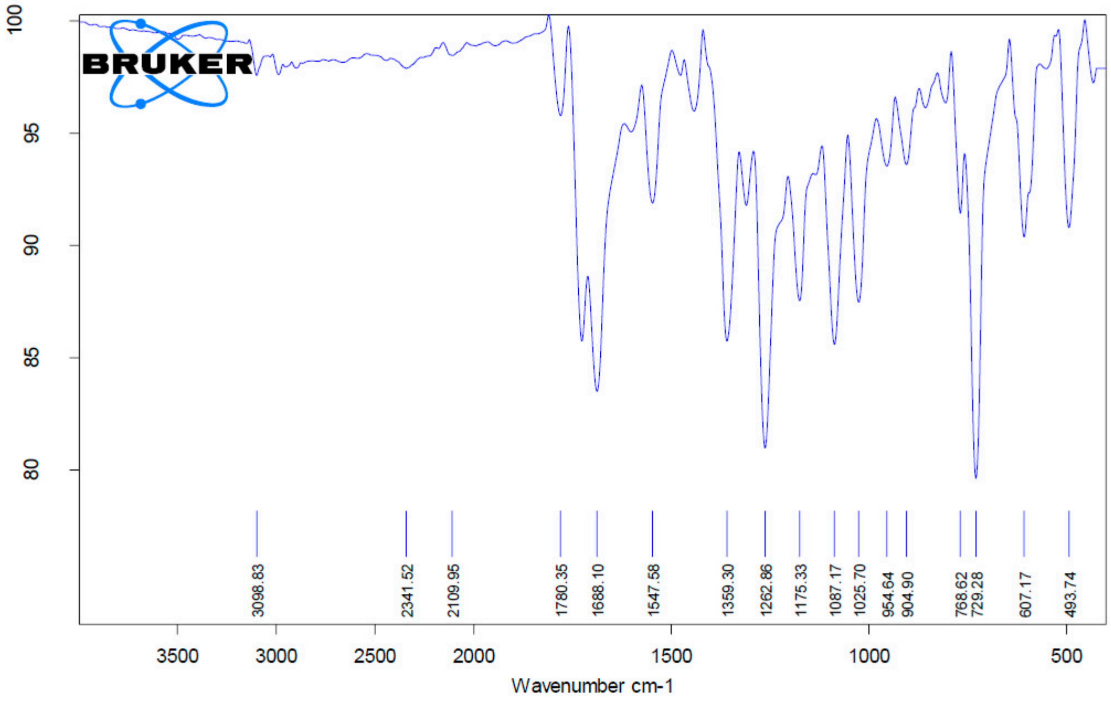

Experimental IR spectrum of compound 7

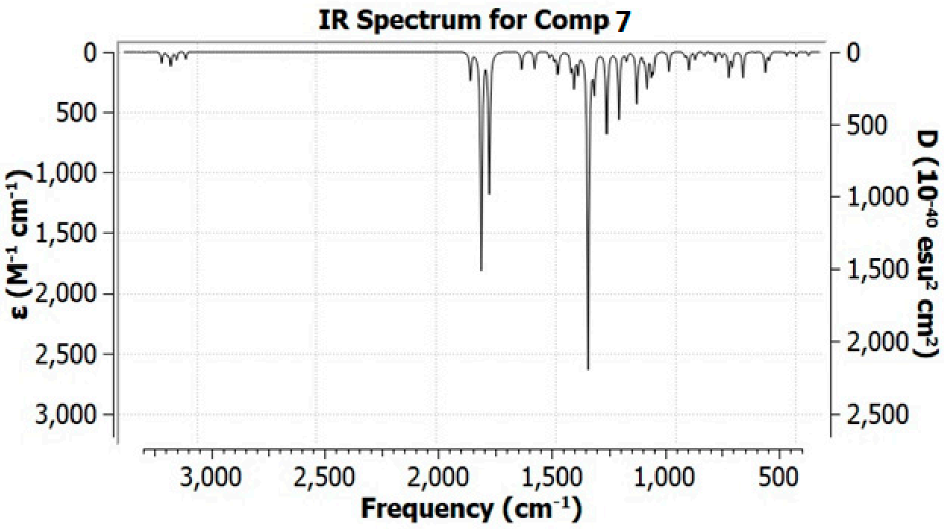

Predicted IR spectrum of compound 7

Supporting NMR spectra for the targets compounds

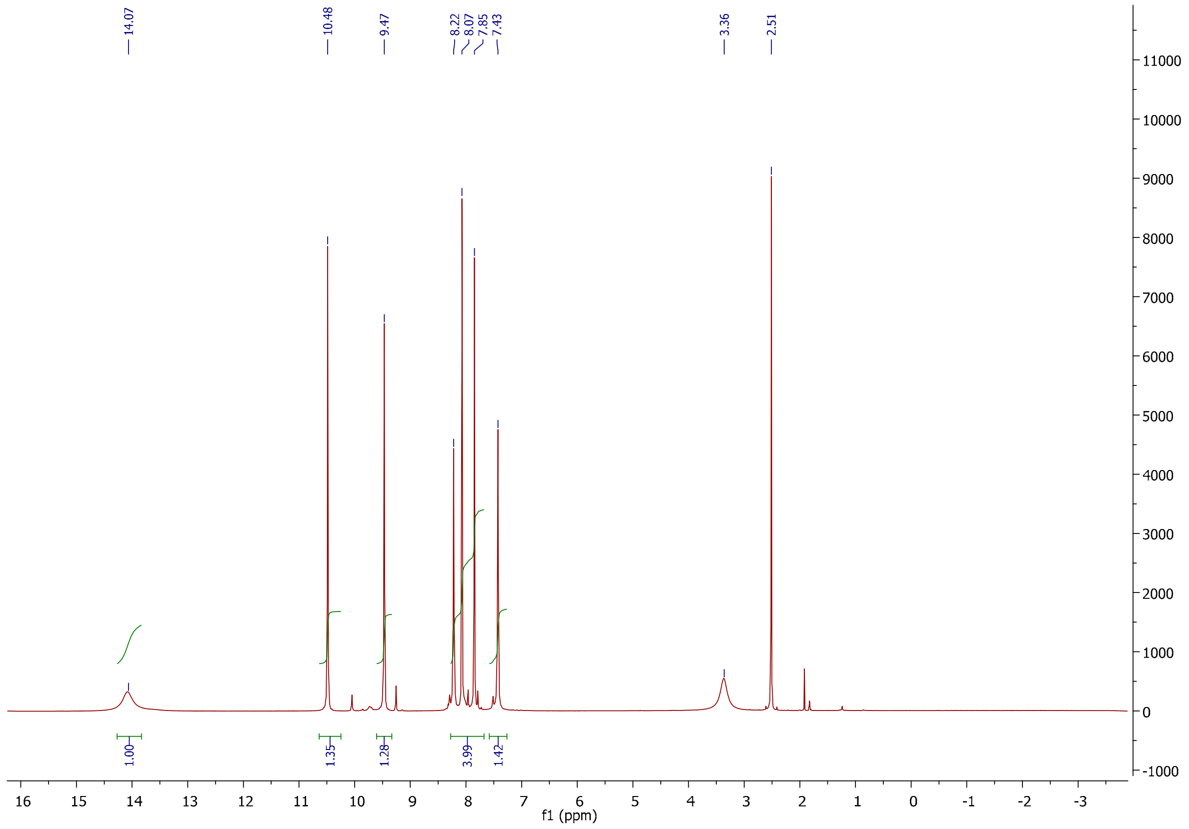

^{1}H NMR spectrum (700 MHz, DMSO-d_6) of compound **1**

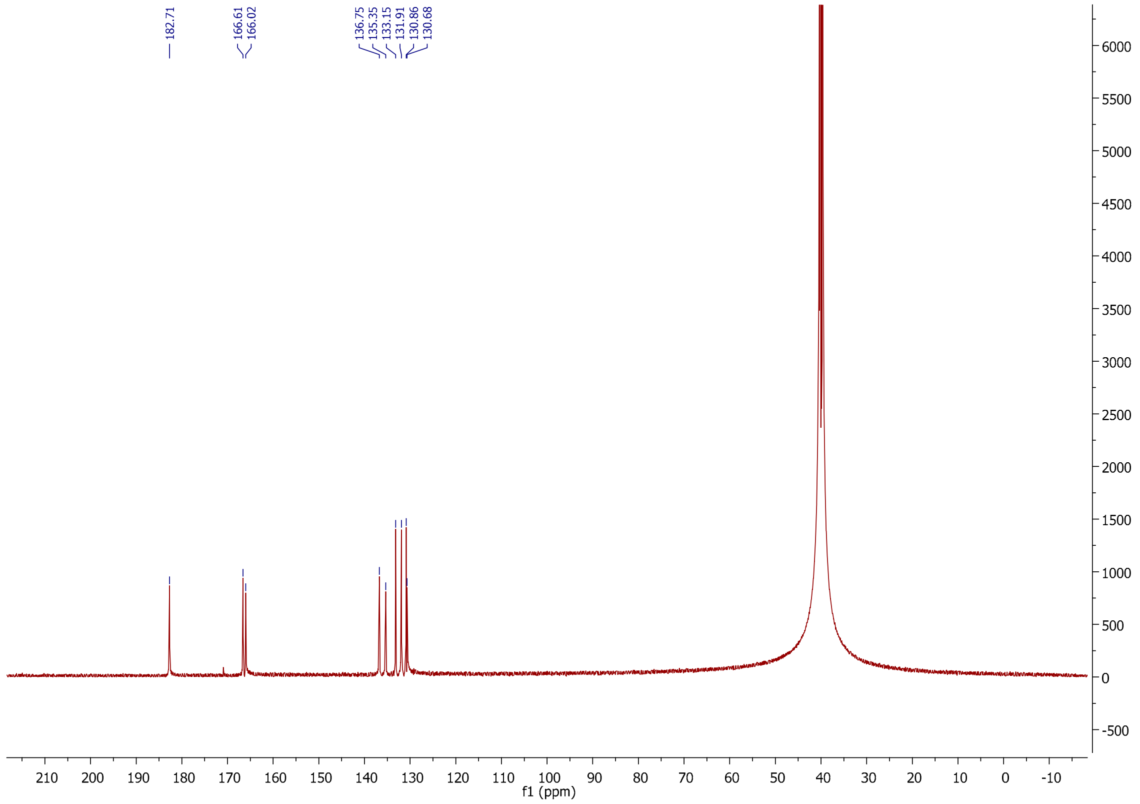

^{13}C NMR spectrum (175 MHz, DMSO-d_6) of compound **1**

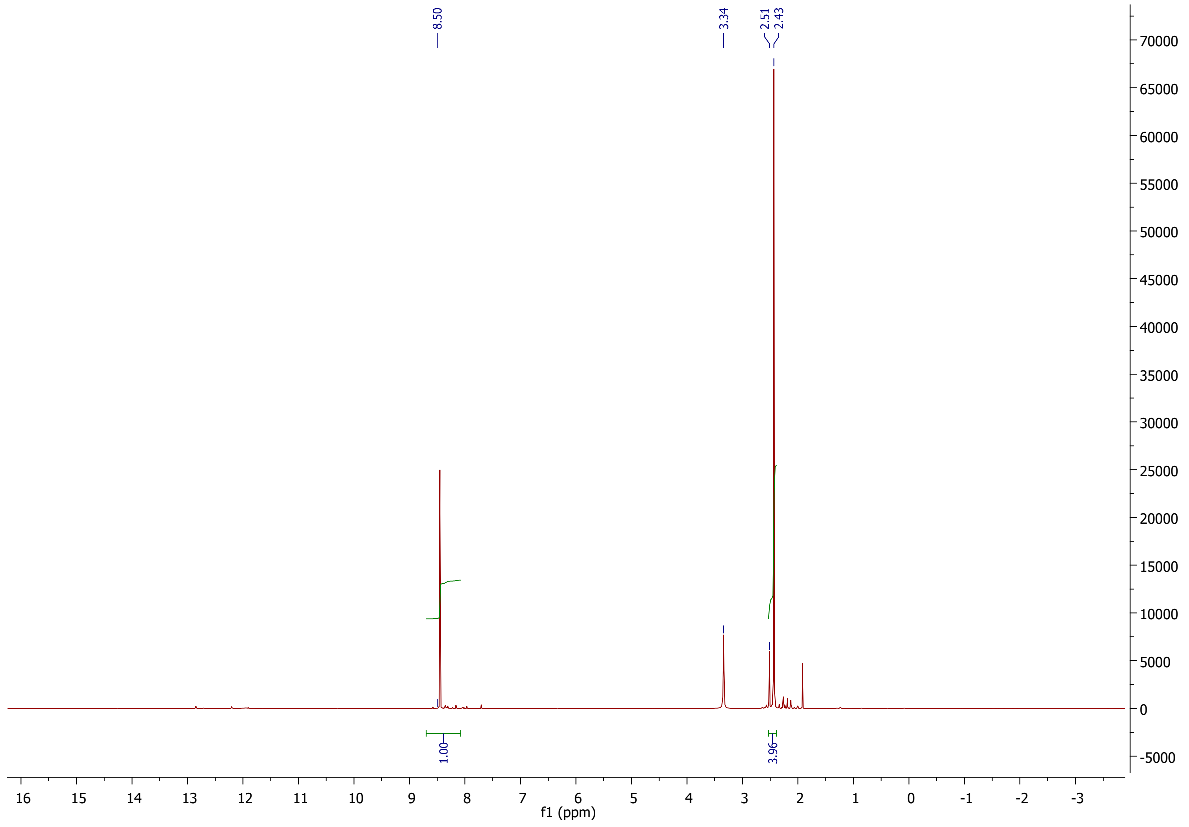

^{1}H NMR spectrum(700 MHz, DMSO-d_6) of compound **2**

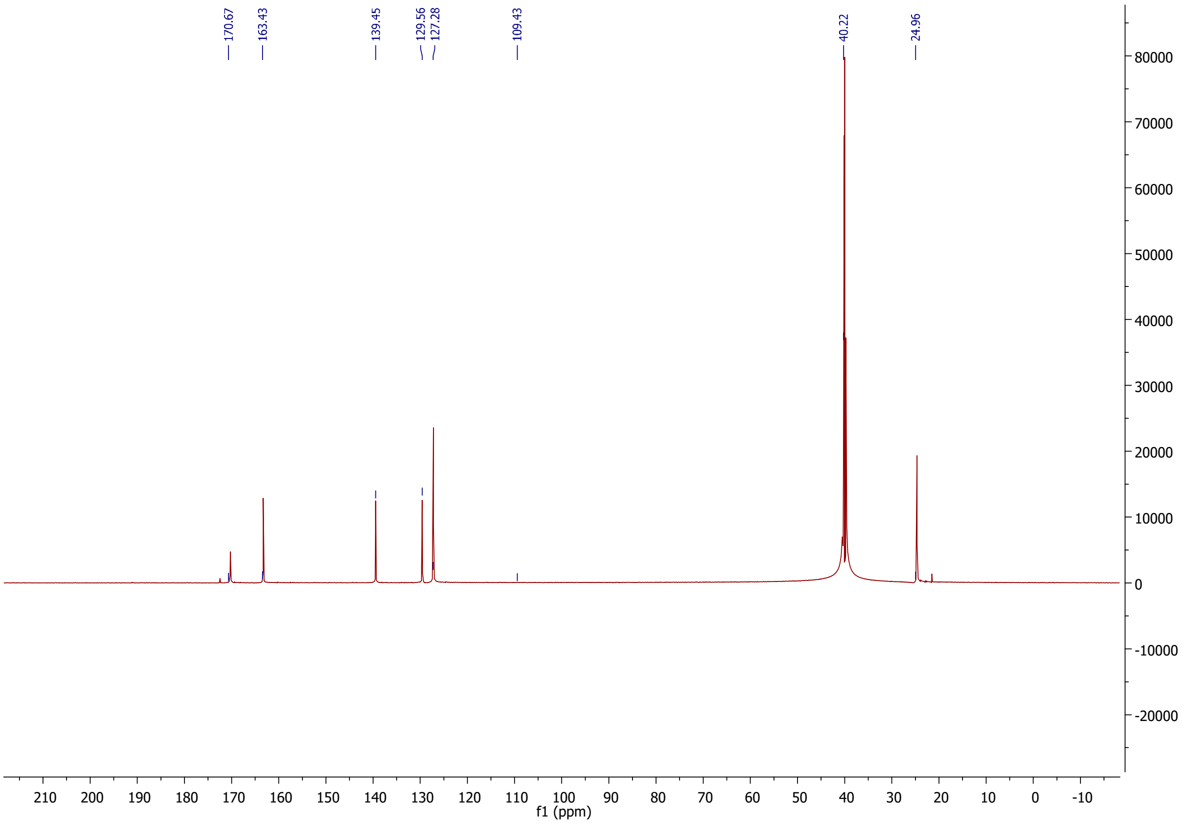

^{13}C NMR spectrum(175 MHz, DMSO-d_6) of compound **2**

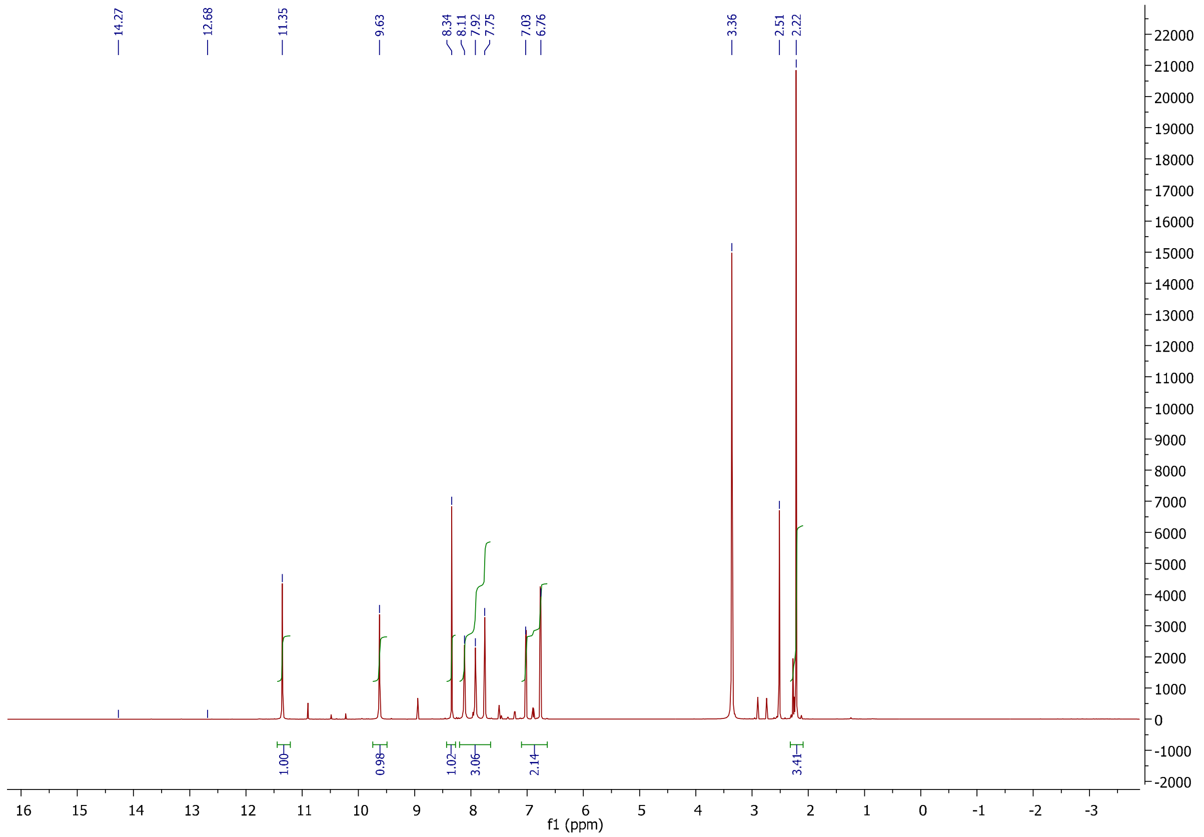

^{1}H NMR spectrum(700 MHz, DMSO-d_6) of compound **5**

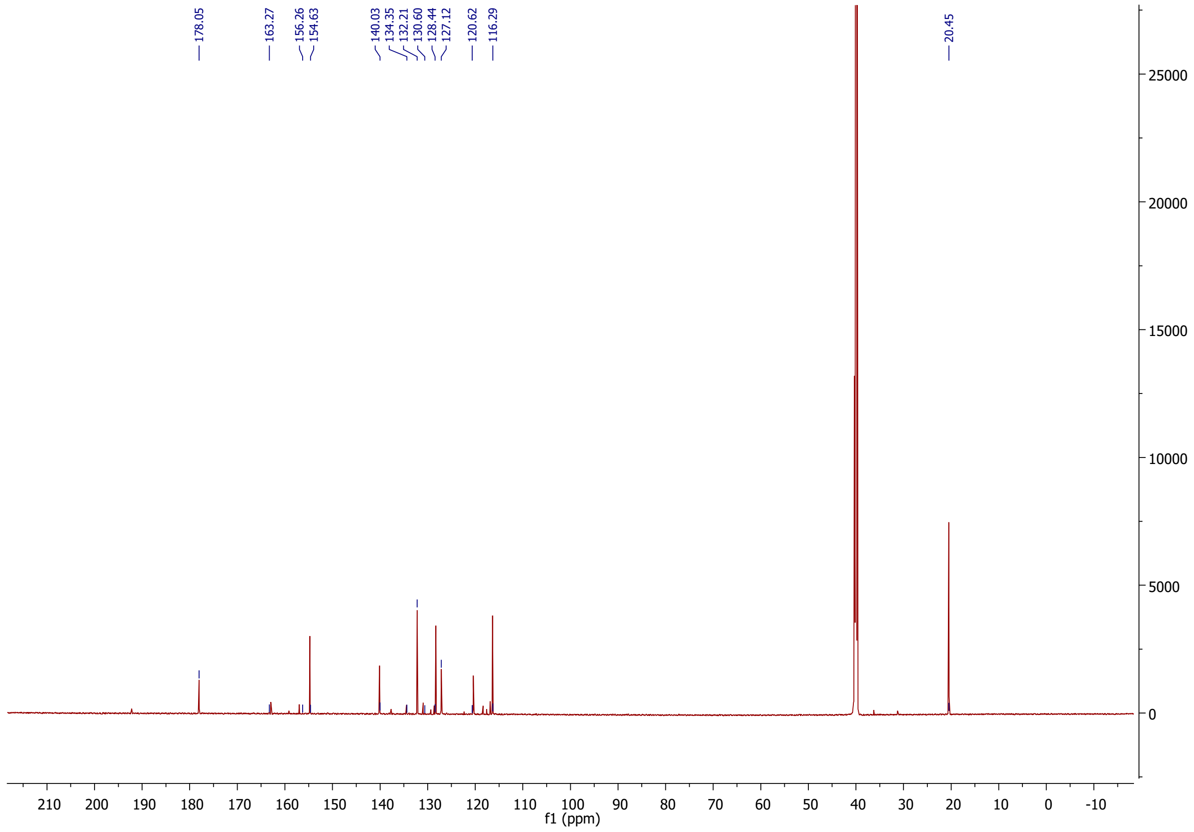

^{13}C NMR spectrum(175 MHz, DMSO-d_6) of compound **5**

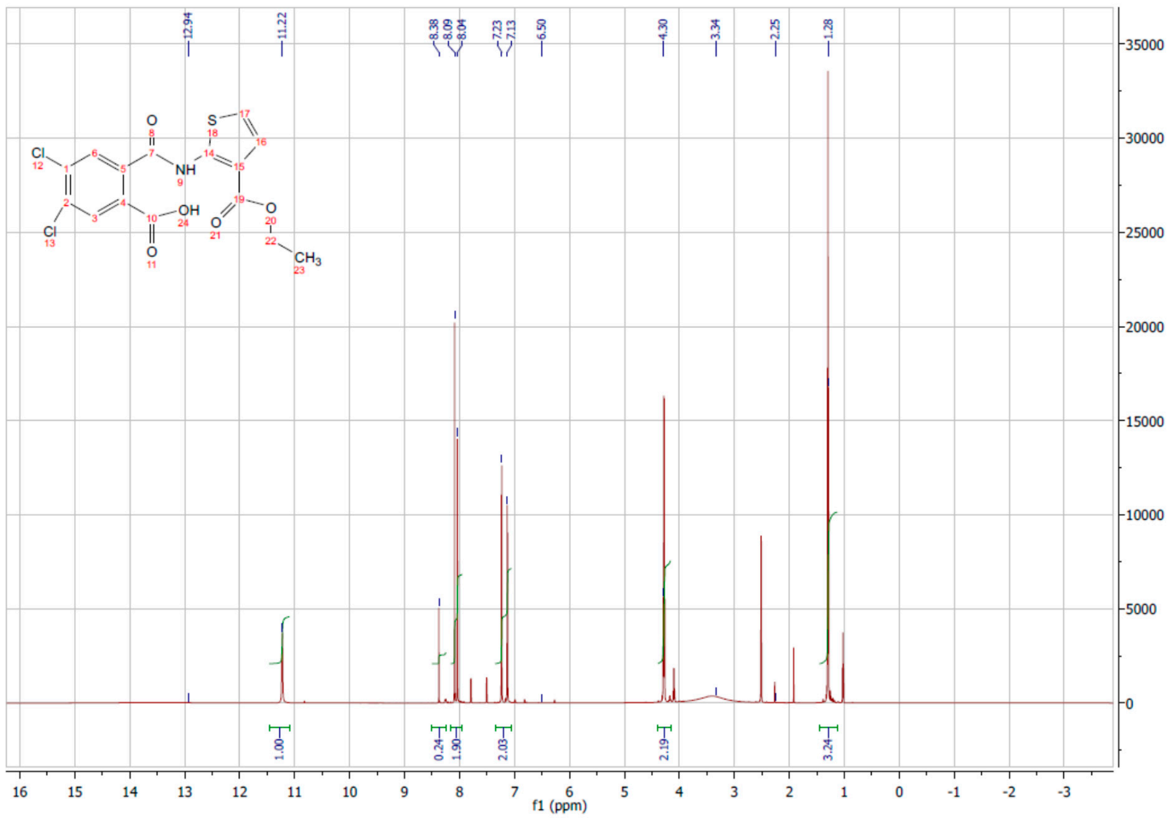

^{1}H NMR spectrum (700 MHz, DMSO-d_6) of compound **6**

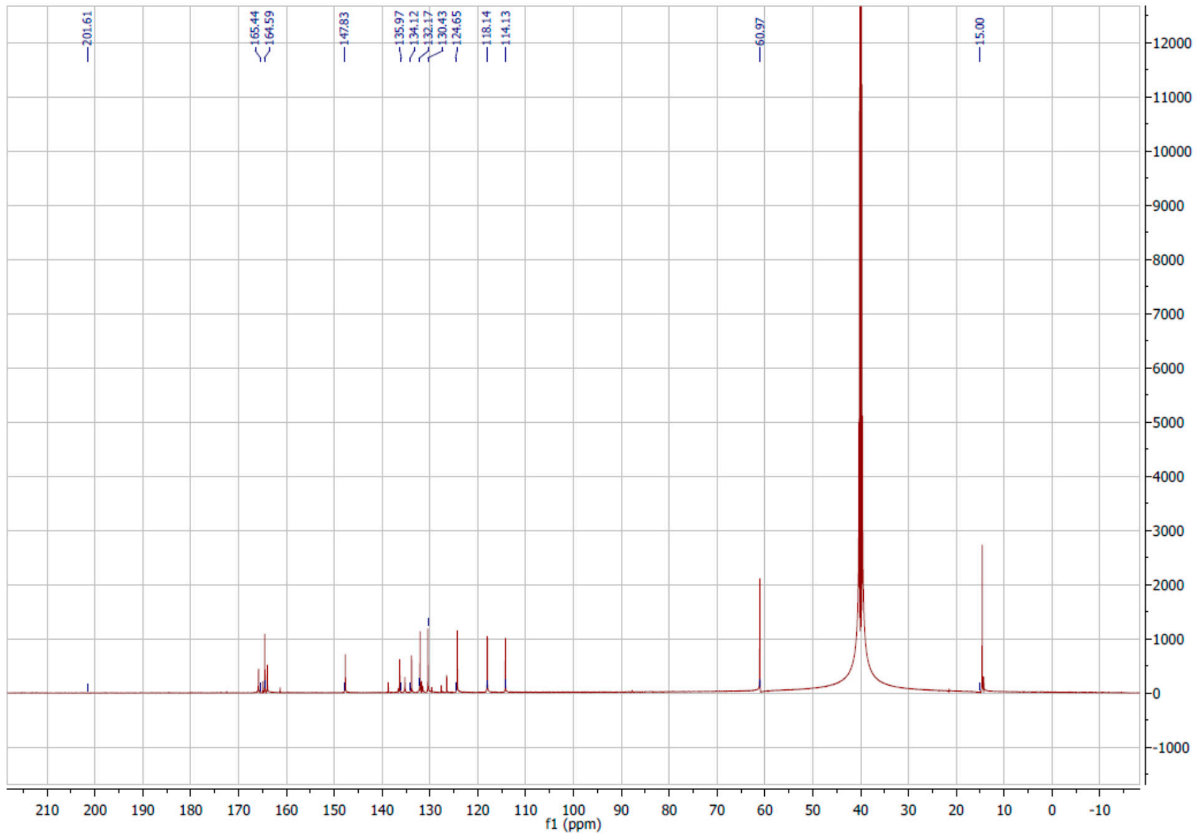

^{13}C NMR spectrum (175 MHz, DMSO-d_6) of compound **6**

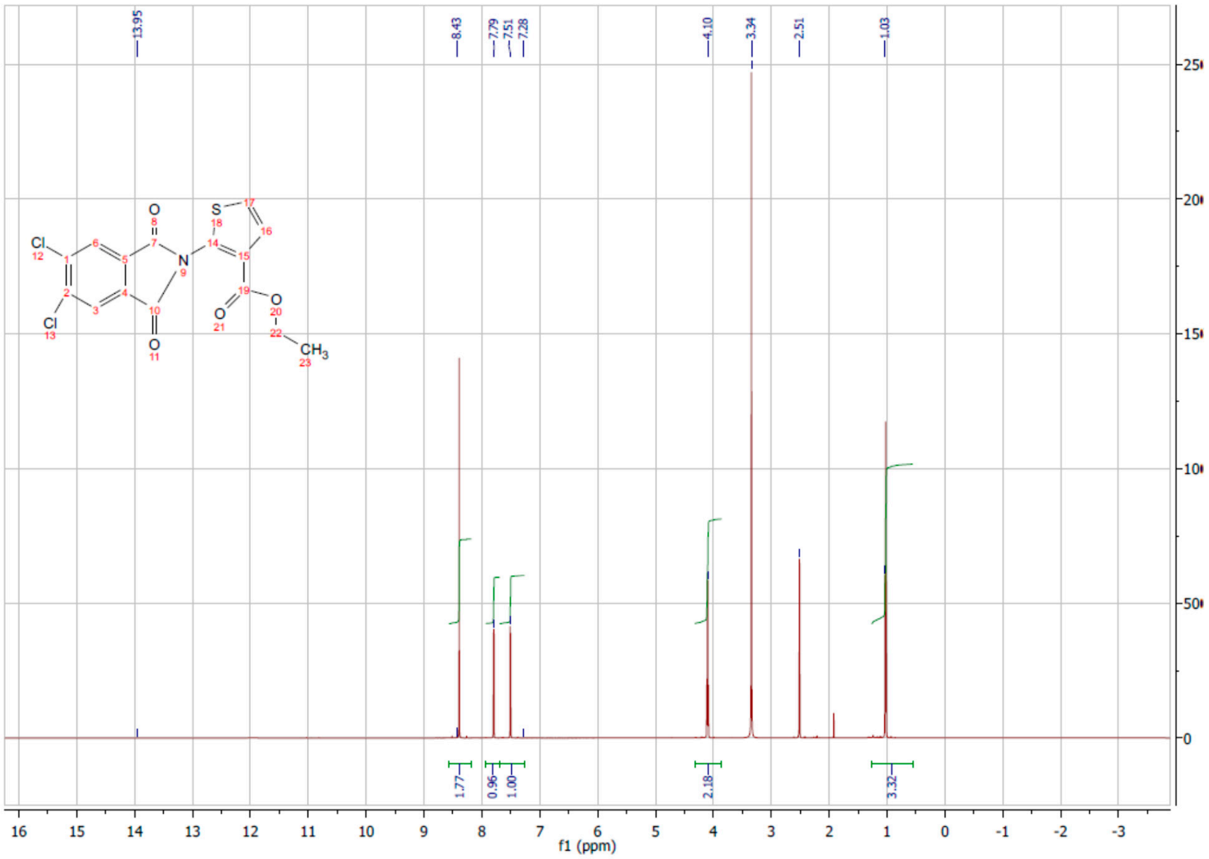

^{1}H NMR spectrum (700 MHz, DMSO-d_6) of compound **7**

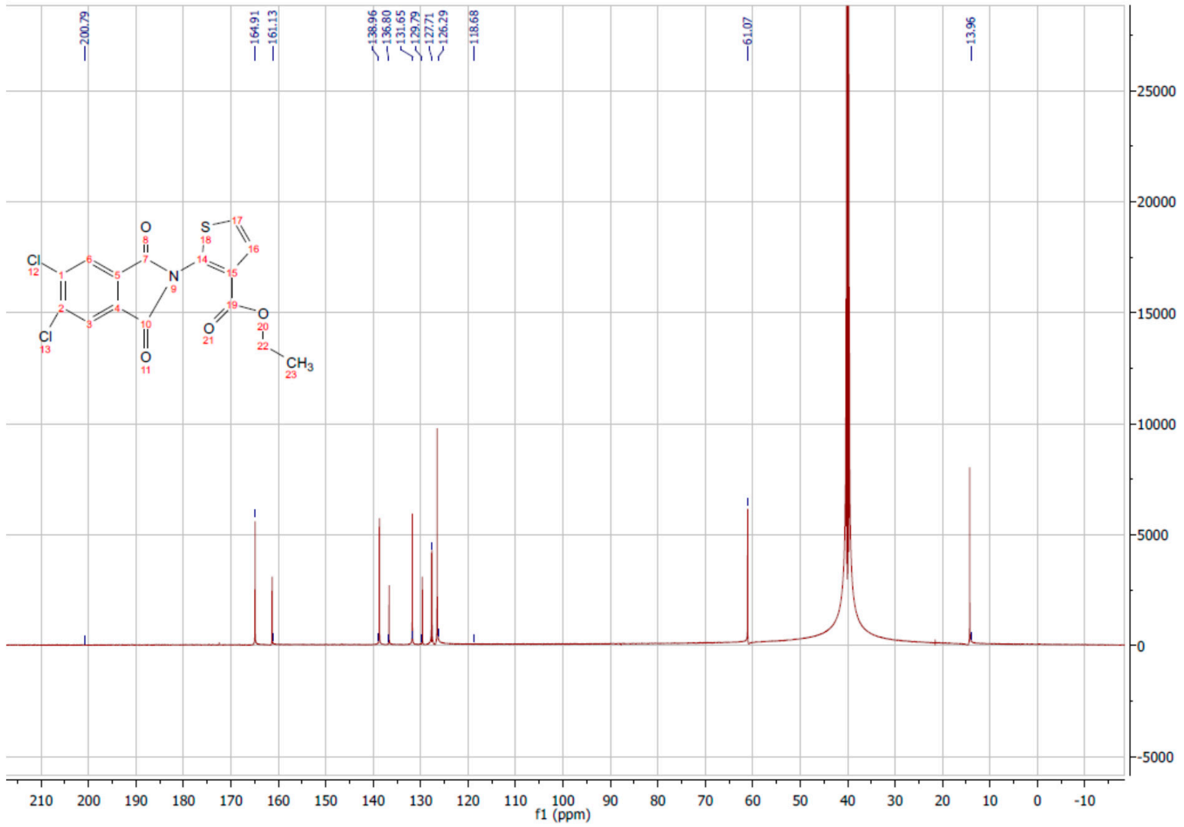

^{13}C NMR spectrum (175 MHz, DMSO-d_6) of compound **7**

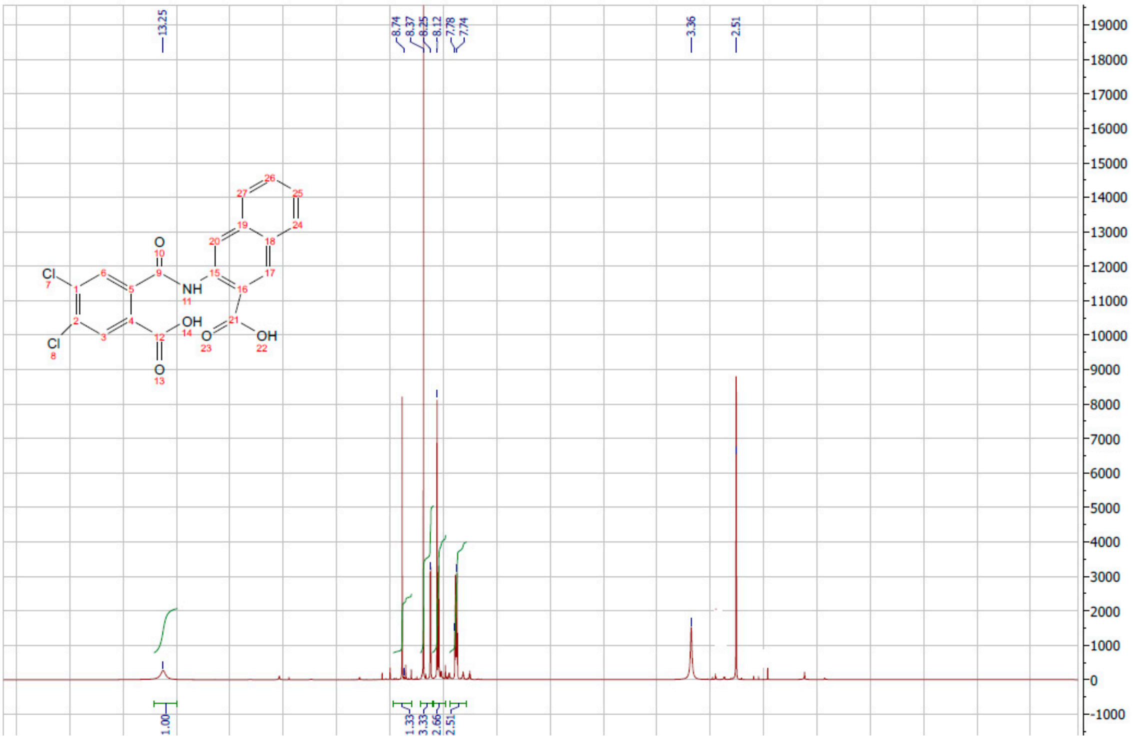

^{1}H NMR spectrum (700 MHz, DMSO-d_6) of compound **12**

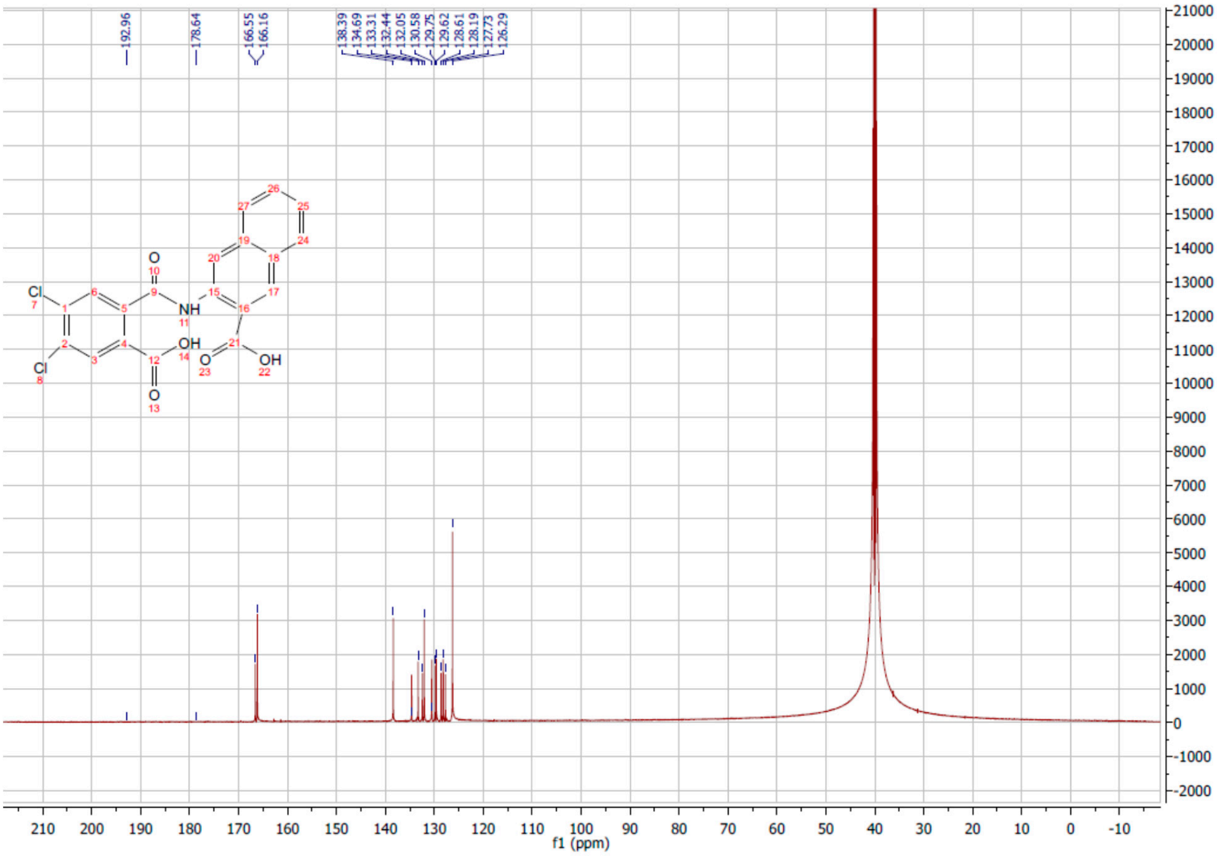

^{13}C NMR spectrum (175 MHz, DMSO-d_6) of compound **12**

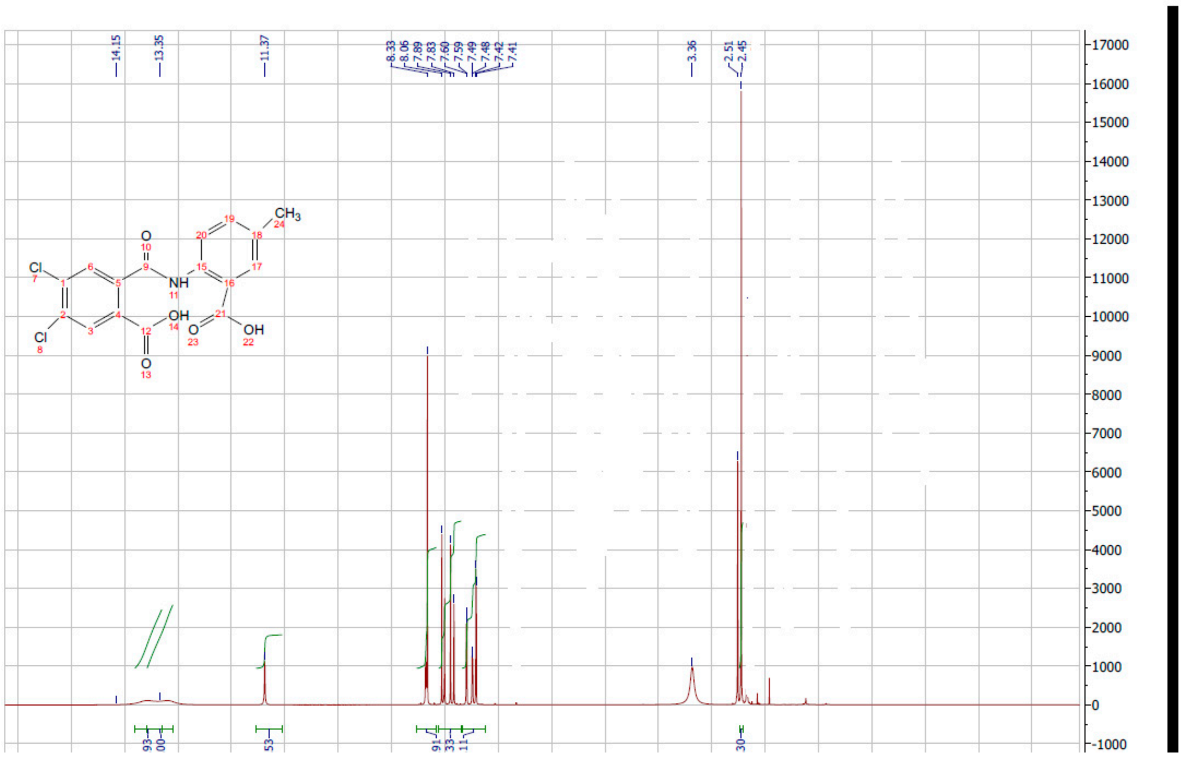

^{1}H NMR spectrum (700 MHz, DMSO-d_6) of compound **11**

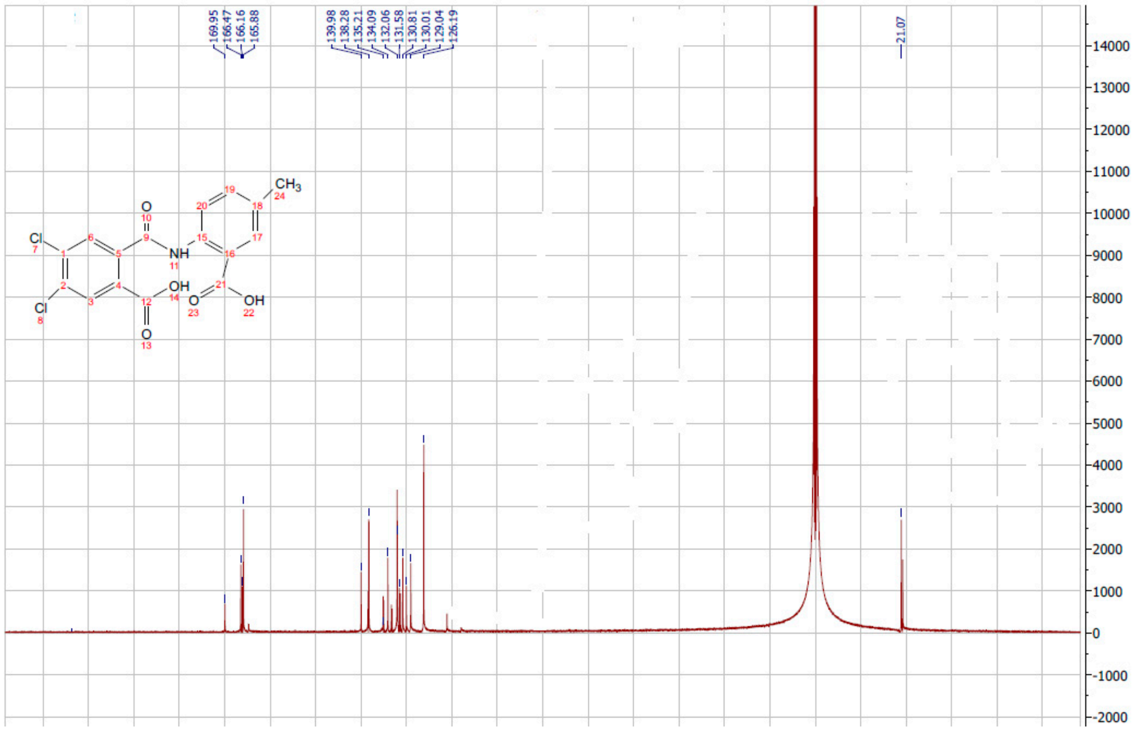

^{13}C NMR spectrum (175 MHz, DMSO-d_6) of compound **11**

Supporting EI-HRMS spectra for compounds 6 and 7

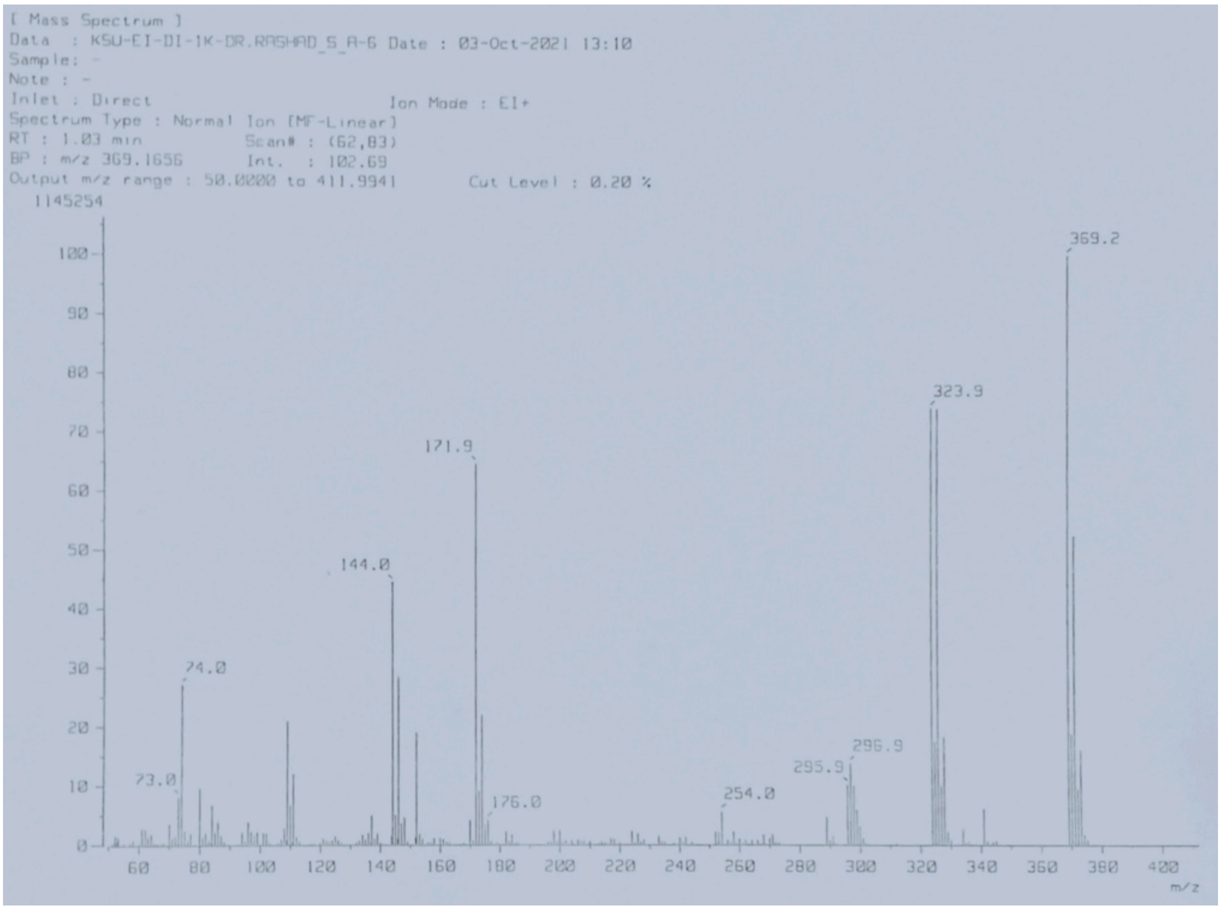

EI-HRMS spectrum of compound 7

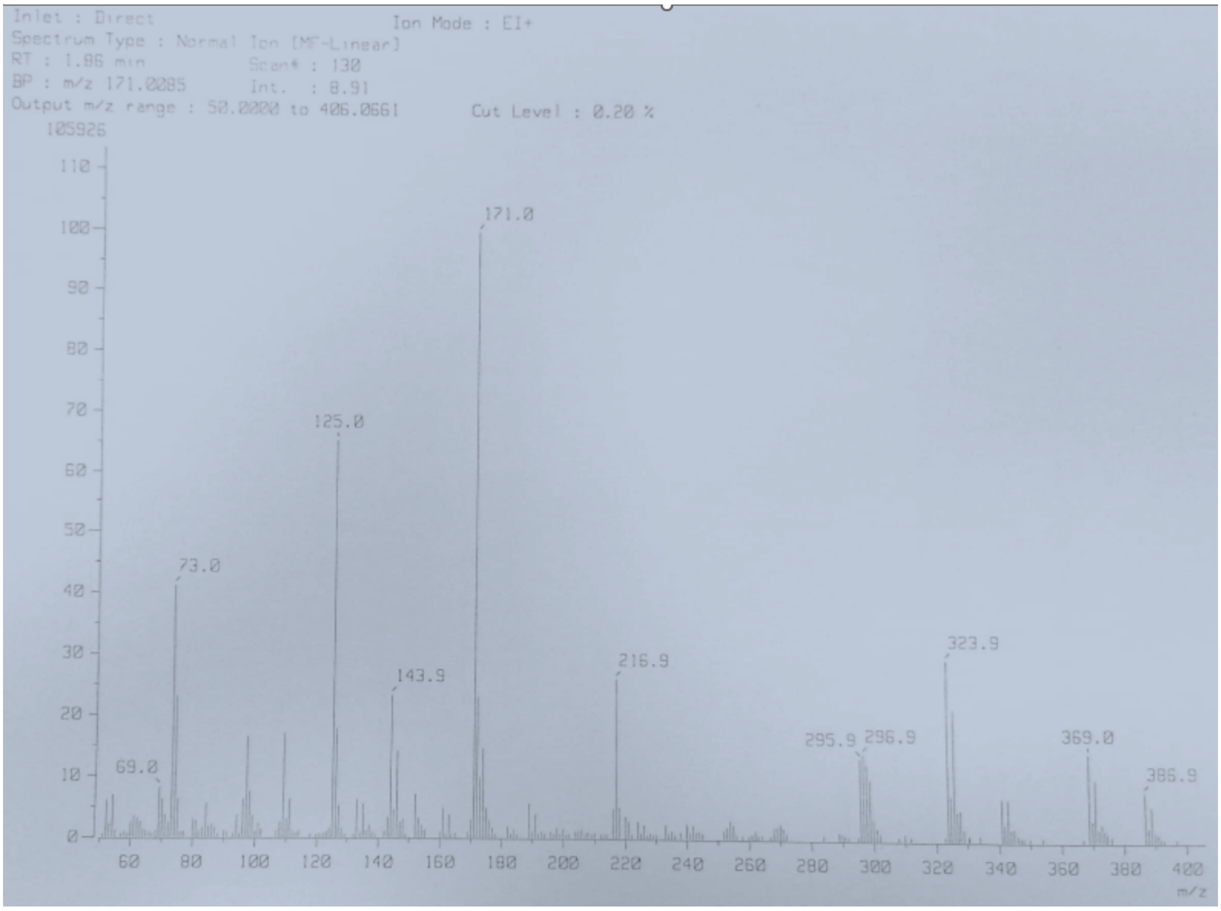

EI-HRMS of compound 6

Table S1. Values of the Fukui function of the compound **2.**

Atom	q(N)	q (N+1)	q (N-1)	f-	f+	f^0	CDD	Electrophilicity	Nucleophilicity	s-	s+	s0	s+/s-	s-/s+
C 1	0.0302	-0.026	0.0718	0.0416	0.0562	0.0489	0.0146	0.1006	0.0618	0.1419	0.1919	0.1669	1.3523	0.7395
C 2	-0.0169	-0.0508	0.0078	0.0247	0.0339	0.0293	0.0092	0.06061	0.03665	0.0842	0.1156	0.0999	1.3739	0.7279
C 3	-0.012	-0.0668	0.0234	0.0354	0.0548	0.0451	0.0194	**0.09807**	0.0526	0.1208	0.1871	0.1539	1.5488	0.6457
C 4	-0.0143	-0.0699	0.0212	0.0355	0.0556	0.0455	0.0201	**0.09943**	0.05278	0.1212	0.1897	0.1554	1.565	0.639
C 5	-0.0181	-0.0537	0.0089	0.027	0.0356	0.0313	0.0086	0.06374	0.04014	0.0922	0.1216	0.1069	1.3192	0.7581
C 6	0.0292	-0.0233	0.0753	0.0461	0.0525	0.0493	0.0064	0.0939	0.06848	0.1573	0.1791	0.1682	1.139	0.878
H 7	0.0675	0.0415	0.0882	0.0207	0.026	0.0233	0.0053	0.04648	0.03077	0.0706	0.0887	0.0797	1.2548	0.7969
H 8	0.0663	0.0401	0.0882	0.0219	0.0262	0.0241	0.0044	0.04695	0.03254	0.0747	0.0896	0.0821	1.1987	0.8342
Cl 9	-0.0186	-0.1137	0.1151	0.1337	**0.0951**	**0.1144**	-0.0386	**0.1701**	0.19868	0.4562	0.3245	0.3903	0.7112	1.4061
Cl 10	-0.0174	-0.1151	0.1099	0.1272	**0.0977**	**0.1125**	-0.0295	**0.1748**	0.18911	0.4342	0.3334	0.3838	0.7679	1.3023
C 11	-0.0863	-0.0973	-0.0548	0.0315	0.011	0.0212	-0.0206	0.01962	0.04686	0.1076	0.0374	0.0725	0.3478	2.8748
H 12	0.0505	0.0408	0.0745	0.024	0.0096	0.0168	-0.0144	0.01722	0.03565	0.0819	0.0328	0.0574	0.4011	2.493
H 13	0.0501	0.0274	0.0749	0.0248	0.0227	0.0237	-0.0021	0.04057	0.03684	0.0846	0.0774	0.081	0.9148	1.0931
H 14	0.0469	0.0388	0.0723	0.0254	0.0081	0.0168	-0.0173	0.0145	0.03781	0.0868	0.0277	0.0572	0.3185	3.1398
C 15	0.1992	0.1874	0.2432	0.0441	0.0117	0.0279	-0.0324	0.02097	0.06551	0.1504	0.04	0.0952	0.2659	3.7612
O 16	-0.229	-0.2687	-0.0873	**0.1417**	0.0396	0.0907	-0.1021	0.07088	0.21065	0.4837	0.1352	0.3094	0.2795	3.5777
C 17	0.1845	0.1176	0.2082	0.0237	0.0669	0.0453	0.0432	**0.11971**	0.03525	0.0809	0.2283	0.1546	2.821	0.3545
C 18	0.1819	0.1075	0.1974	0.0155	0.0744	0.0449	0.0589	**0.13308**	0.02306	0.0529	0.2538	0.1534	4.7949	0.2086
O 19	-0.2187	-0.3235	-0.1378	0.0808	**0.1048**	0.0928	0.0239	**0.18745**	0.12014	0.2759	0.3576	0.3167	1.2961	0.7716
O 20	-0.2315	-0.3368	-0.1844	0.0471	**0.1053**	0.0762	0.0582	**0.18831**	0.06998	0.1607	0.3592	0.2599	2.2354	0.4473
N 21	-0.0433	-0.0556	-0.0158	0.0275	0.0123	0.0199	-0.0152	0.02208	0.04089	0.0939	0.0421	0.068	0.4485	2.2297

Table S2. Values of the Fukui function of the compound **5.**

atom	q(N)	q(N+1)	q(N-1)	f-	f+	f0	CDD	Electrophilicity	Nucleophilicity	s-	s+	s0	s+/s-	s-/s+
C 1	0.0264	0.0039	0.0382	0.0118	0.0225	0.0171	0.0107	0.03833	0.03721	0.0532	0.1013	0.0773	1.9046	0.525
C 2	-0.0265	-0.0444	-0.0164	0.0101	0.0179	0.014	0.0078	0.0305	0.03185	0.0455	0.0806	0.0631	1.7705	0.5648
C 3	-0.0023	-0.0227	-0.0085	-0.0062	0.0204	0.0071	0.0266	0.03476	-0.01957	-0.028	0.0919	0.032	-3.2838	-0.3045
C 4	-0.0066	-0.0243	-0.0021	0.0045	0.0178	0.0111	0.0133	0.03032	0.01407	0.0201	0.0801	0.0501	3.9826	0.2511
C 5	-0.0305	-0.0451	-0.018	0.0125	0.0146	0.0136	0.0021	0.02492	0.03941	0.0563	0.0659	0.0611	1.169	0.8554
C 6	0.0237	-0.0063	0.0438	0.0201	0.03	0.0251	0.0099	0.05115	0.06343	0.0907	0.1352	0.1129	1.491	0.6707
H 7	0.0553	0.0438	0.0619	0.0066	0.0115	0.009	0.0049	0.01958	0.02075	0.0297	0.0517	0.0407	1.7443	0.5733
H 8	0.0557	0.0446	0.0655	0.0098	0.0112	0.0105	0.0014	0.01902	0.03083	0.0441	0.0503	0.0472	1.1407	0.8766
C 9	-0.0237	-0.077	0.0259	0.0495	0.0533	0.0514	0.0038	0.09092	0.15615	0.2233	0.2403	0.2318	1.0764	0.929
C 10	-0.022	-0.0707	0.0167	0.0387	0.0487	0.0437	0.01	0.08302	0.12195	0.1744	0.2194	0.1969	1.2586	0.7946
C 11	0.1524	0.1235	0.1789	0.0265	0.0288	0.0277	0.0023	0.04911	0.08363	0.1196	0.1298	0.1247	1.0857	0.921
C 12	0.2165	0.2032	0.2151	-0.0013	0.0133	0.006	0.0146	0.02267	-0.00423	-0.006	0.0599	0.0269	-9.9082	-0.1009
O 13	-0.2835	-0.3261	-0.2304	0.0531	0.0426	0.0478	-0.0105	0.0726	0.1674	0.2393	0.1919	0.2156	0.8018	1.2472
O 14	-0.2452	-0.2708	-0.228	0.0172	0.0257	0.0214	0.0084	0.04374	0.05423	0.0775	0.1156	0.0966	1.4909	0.6707
N 15	-0.0386	-0.0415	0.0206	0.0592	0.0029	0.031	-0.0563	0.00489	0.18661	0.2668	0.0129	0.1399	0.0484	20.6581
O 16	-0.1395	-0.1465	-0.1415	-0.002	0.007	0.0025	0.009	0.01198	-0.00626	-0.009	0.0317	0.0114	-3.5376	-0.2827

H 17	0.1765	0.1716	0.1792	0.0027	0.0049	0.0038	0.0022	0.00836	0.00864	0.0123	0.0221	0.0172	1.7887	0.5591
C 18	0.083	0.0538	0.1088	0.0258	0.0291	0.0275	0.0034	0.04968	0.08125	0.1162	0.1313	0.1237	1.1305	0.8846
N 19	-0.1487	-0.1851	-0.152	-0.0033	0.0364	0.0166	0.0397	0.06212	-0.01039	-0.0149	0.1642	0.0747	-11.05	-0.0905
C 20	0.0738	-0.0001	0.093	0.0191	**0.074**	0.0466	0.0548	0.12613	0.06034	0.0863	0.3334	0.2098	3.8646	0.2588
H 21	0.0416	0.0157	0.0526	0.011	0.0258	0.0184	0.0148	0.04407	0.03475	0.0497	0.1165	0.0831	2.3448	0.4265
S 22	-0.2803	-0.416	0.0619	**0.3422**	0.1358	0.239	-0.2064	0.23151	**1.0787**	1.5423	0.6119	1.0771	0.3968	2.5204
N 23	-0.0109	-0.0274	0.0669	0.0778	0.0165	0.0472	-0.0614	0.02808	0.24539	0.3508	0.0742	0.2125	0.2116	4.7263
H 24	0.1392	0.1234	0.1657	0.0265	0.0158	0.0211	-0.0108	0.02688	0.08357	0.1195	0.071	0.0953	0.5945	1.682
H 25	0.0909	0.0885	0.1071	0.0163	0.0024	0.0093	-0.0139	0.00401	0.05133	0.0734	0.0106	0.042	0.1444	6.9231
C 26	-0.027	-0.0395	-0.0254	0.0016	0.0126	0.0071	0.011	0.02146	0.00506	0.0072	0.0567	0.032	7.8355	0.1276
C 27	-0.0356	-0.0617	-0.0311	0.0044	0.0261	0.0153	0.0217	0.04456	0.014	0.02	0.1178	0.0689	5.8857	0.1699
C 28	0.0842	0.0595	0.1011	0.0169	0.0247	0.0208	0.0077	0.04205	0.05336	0.0763	0.1111	0.0937	1.4569	0.6864
C 29	-0.0014	-0.0189	0.0134	0.0149	0.0174	0.0162	0.0026	0.02975	0.04684	0.067	0.0786	0.0728	1.1742	0.8516
H 30	0.0414	0.0308	0.0425	0.0012	0.0106	0.0059	0.0094	0.01799	0.00366	0.0052	0.0476	0.0264	9.0778	0.1102
C 31	-0.064	-0.0938	-0.0466	0.0174	0.0298	0.0236	0.0124	0.05085	0.05482	0.0784	0.1344	0.1064	1.7151	0.5831
C 32	-0.0261	-0.077	0.0034	0.0295	0.0509	0.0402	0.0213	0.08677	0.09313	0.1331	0.2293	0.1812	1.7225	0.5805
H 33	0.0464	0.0252	0.0608	0.0143	0.0212	0.0178	0.0069	0.03614	0.04512	0.0645	0.0955	0.08	1.4807	0.6754
H 34	0.0506	0.0249	0.067	0.0164	0.0257	0.021	0.0093	0.0438	0.05165	0.0739	0.1158	0.0948	1.5675	0.638
O 35	-0.169	-0.1859	-0.156	0.013	0.0168	0.0149	0.0038	0.02871	0.04098	0.0586	0.0759	0.0672	1.2949	0.7723
H 36	0.1911	0.1747	0.2036	0.0125	0.0164	0.0145	0.0039	0.02799	0.03939	0.0563	0.074	0.0652	1.3137	0.7612
C 37	-0.0821	-0.0899	-0.076	0.0061	0.0078	0.007	0.0017	0.01335	0.01921	0.0275	0.0353	0.0314	1.2849	0.7783
H 38	0.0362	0.031	0.0392	0.0031	0.0052	0.0041	0.0021	0.00887	0.00966	0.0138	0.0234	0.0186	1.6973	0.5892
H 39	0.0375	0.0249	0.0477	0.0101	0.0126	0.0114	0.0025	0.0215	0.03189	0.0456	0.0568	0.0512	1.2465	0.8022
H 40	0.0408	0.0274	0.0511	0.0103	0.0135	0.0119	0.0032	0.02298	0.03244	0.0464	0.0607	0.0536	1.3094	0.7637

Table S3. Experimental and calculated ^{13}C& ^{1}H isotropic chemical shifts (ppm) for the compound **5.**

Experimental (^{13}C)	Calculated	Experimental(^{1}H)	Calculated
178.05	188.949	12.68	11.6345
	171.5843	11.35	9.6157
163.27	169.4083	9.63	9.1159
157.32	164.681	8.34	8.5766
156.26	154.9012	8.11	8.0602
154.63	148.5468	7.92	7.7496
	148.0065	7.75	7.654
140.03	144.2204	7.03	7.0914
138.2	139.2031	6.76	6.9679
134.35	139.1244		4.9127
132.21	138.2221	2.21	2.381233
130.6	132.7321		
128.44	132.2819		
127.12	131.2099		
120.62	125.3442		
116.29	120.2454		
20.45	19.6065		

Table S4. Experimental and calculated ^{13}C& ^{1}H isotropic chemical shifts (ppm) for the compound **2.**

Experimental (^{1}H)	Calculated	Experimental(^{13}C)	Calculated
8.5	8.1548	170.67	170.0037
8.5	8.119	163.43	167.5396
			151.8524
2.43	2.8176		133.7365
		139.45	133.0332
		129.58	130.4224
		127	130.2556
		24	27.4899
